# The mushrooms on the menu (MOM) study: vitamin D mushrooms (UV-exposed) are a feasible and acceptable way to increase vitamin D intake in a residential aged care facility

**DOI:** 10.3389/fpubh.2025.1568202

**Published:** 2025-06-18

**Authors:** Celeste Ferraris, Michelle Blumfield, Emily Duve, Lucy Downey, Jutta Wright, Saamia Khan, Emma L. Beckett, Flávia Fayet-Moore

**Affiliations:** ^1^FOODiQ Global, Sydney, NSW, Australia; ^2^School of Environmental and Life Sciences, The University of Newcastle, Ourimbah, NSW, Australia

**Keywords:** vitamin D, aged care, UV-exposed mushrooms, diet, food service, food-first approach

## Abstract

**Background:**

Vitamin D deficiency is highly prevalent in aged care due to reduced endogenous synthesis of vitamin D and less time outdoors, and is associated with poorer health outcomes. Supplementation implementation is variable and dosages are often suboptimal. Due to limited food sources, diet is frequently overlooked, yet mushrooms can raise vitamin D levels in deficient individuals similar to a supplement.

**Objectives:**

Mushrooms on the Menu (MOM) was a 10-week prospective pre-post mixed method study that evaluated the feasibility of adding vitamin D mushrooms to the menu of a residential aged care facility.

**Methods:**

During the 4-week baseline phase, residential care (RC) participants ordered meals from the standard food service menu, while independent living (IL) followed their usual diet. During the 4-week MOM phase, participants were instructed to consume at least one mushroom meal containing 75 g of UV-exposed mushrooms daily. In RC, 26 recipes were modified and two recipes newly created to include mushrooms. RC participants chose a minimum of one mushroom meal from the lunch or dinner menu, and IL residents were instructed to prepare at least one mushroom meal daily. Dietary intakes were estimated by plate wastage (RC) or 24-h recalls (IL), while qualitative data were collected during and post the MOM.

**Results:**

In RC (*n* = 60), vitamin D provision via mushrooms from the MOM menu increased by 180% compared to the standard menu (7.0 vs. 2.5 μg, *p* < 0.0001), with no significant differences in total energy and other nutrients. During MOM, vitamin D intake increased by 212% for RC (6.0 vs. 18.7 μg; *p* < 0.0001) and 740% for IL participants (*n* = 12; 8.7 vs. 73.1 μg; *p* < 0.0001) compared to baseline, representing 125 and 1,387% of the adequate intake (AI) for over 70-year-old’s, respectively. Over 75% of participants rated the taste of vitamin D mushroom meals as good or excellent, while qualitative data reported participants enjoyed mushrooms as both hero and complimentary ingredients. Over 75% of staff understood the health benefits of vitamin D mushrooms and found the meals easy to prepare, but preferred low-burden ordering and preparation processes. Both participants and staff supported the continued inclusion of MOM.

**Conclusion:**

MOM is a well-accepted food-first approach that provides substantial vitamin D to aged care residents.

## 1 Introduction

Vitamin D deficiency is highly prevalent in aged care (up to 95% worldwide) (1, 2), and is associated with many age-related health conditions, including osteoporosis, increased risk of falls and fractures, cognitive decline, hypertension, type II diabetes, and cancer (1, 3). Residents in aged care are at higher risk of vitamin D deficiency due to limited sunlight exposure, reduced outdoor activity, and physiological changes in vitamin D synthesis with aging (4, 5). Consequently, dietary vitamin D requirements in older adults are up to three times higher than those under 50 years (15 vs. 5 μg/day) to compensate for these factors, yet most aged care Australians have inadequate intake (<5.2 μg/day) (2, 6–9). While daily supplementation has been recommended to increase vitamin D levels in aged care, it is not routinely prescribed and uptake is well below levels considered clinically appropriate for population-based interventions (10, 11). Alternatively, compelling evidence indicates a food-first approach to nutrition may improve nutritional status and health outcomes, reduce healthcare usage and costs, and minimize the risks associated with polypharmacy (12–18). Enriching meals with foods important to aged care residents can improve nutrient intake (19, 20) and provide a more balanced and diverse nutrient profile than supplementation alone (21). Thus, aged care providers have shown an increased interest in adopting a food-first approach to improve residents’ nutritional and clinical status (18, 22).

Dietary vitamin D comprises two forms: D_2_, fungi-derived, and D_3_, animal-derived. Mushrooms contain ergosterol and, when exposed to UV light, produce vitamin D_2_ in a reaction similar to endogenous vitamin D_3_ synthesis in human skin (23). UV exposure increases the vitamin D content of edible mushrooms (24–33) and their consumption has been shown to raise vitamin D levels (34, 35) and help meet international dietary vitamin D targets (10–15 μg/day) (30, 33). In Australia, the vitamin D_2_ content of UV-exposed common white button mushroom (*Agaricus bisporus*) is well above dietary targets at 24.2 μg/100 g (36). Mushrooms provide several micronutrients, including riboflavin, biotin, niacin, pantothenic acid, folate, selenium, phosphorous, chromium and potassium (25, 36), and contain a wide range of health-promoting bioactive compounds (chitin, beta-glucan, glutathione, ergothioneine) (37, 38). They are flavour enhancing, versatile and help support circular farming (39). Consumption of UV-exposed mushrooms can effectively increase vitamin D levels, particularly in individuals with vitamin D insufficiency or deficiency (25, 29, 33). UV-exposed mushrooms have been recommended as a food-based solution to address dietary vitamin D shortfalls (33), positioning mushrooms as an innovative alternative to supplementation.

While food-first interventions in aged care are logical and required, meal provision is complex, and data on the effectiveness of food interventions are required (13). Residential aged care facilities face numerous challenges when implementing dietary interventions, including limited resources, inadequate staffing, and time and economic constraints (18, 40). Few studies have assessed dietary interventions to improve vitamin D intake, with supplementation being the more frequently studied method (19, 41, 42). While it is known that consumption of UV-exposed mushrooms, hereon in termed vitamin D mushrooms, can increase vitamin D status (25, 29, 33), the feasibility and nutritional impact of adding vitamin D mushrooms to the menu of an aged care facility has not been previously investigated. Thus, the aims of this study were to investigate (i) the effect of vitamin D mushrooms (MOM) on vitamin D provision and intake (primary), and (ii) the feasibility of implementing MOM in a residential aged care facility (secondary).

## 2 Materials and methods

### 2.1 Study design

**Figure 1:**
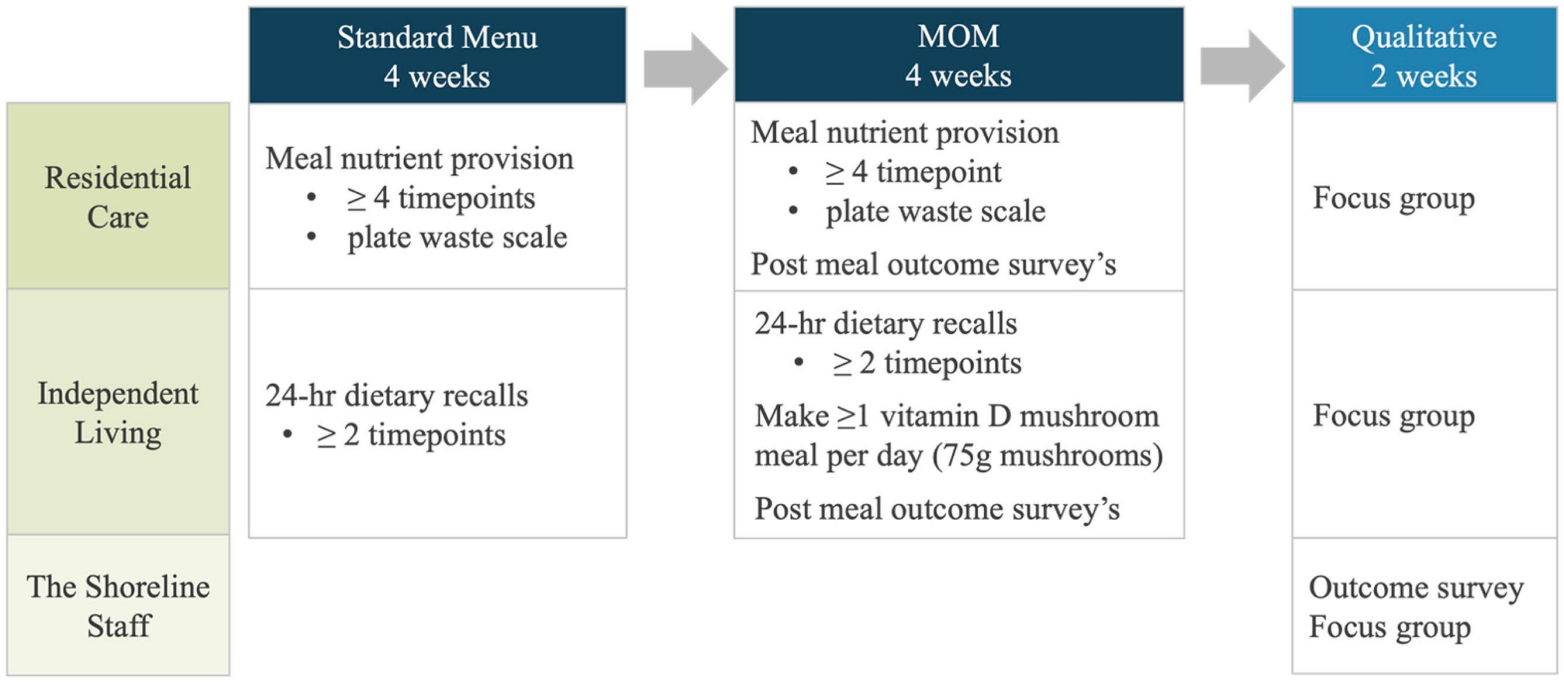
The mushrooms on the menu feasibility study design.

The MOM study is a mixed-method (combining qualitative and quantitative approaches) feasibility study that comprised a pre-post design with quantitative and qualitative methodology (Figure 1). The study was conducted at the residential aged care facility The Shoreline, Coffs Harbour, NSW, Australia between January and May 2024. The Shoreline offers residential care (RC) and independent living (IL) residences. RC residents’ meals are chef-designed, dietitian-approved, and served by staff in dining rooms or private rooms. Residents of IL are free-living and can prepare meals in their apartments or purchase meals from the RC menu.

Based on operational and ethical considerations within the aged care setting, the standard menu (RC) or usual diet (IL) was consumed for 4 weeks, followed by the four-week vitamin D MOM intervention with nutrition provision and intake compared between phases. To capture experiences in eating vitamin D mushroom meals, RC and IL participants completed outcome surveys at randomized times after consuming mushroom meals throughout the intervention. For 2 weeks post-MOM, outcome surveys and focus group interviews with food service and clinical staff were used to gain a qualitative understanding of the feasibility of adding vitamin D mushrooms to the menu. Commercially available vitamin D mushrooms exposed to UV-light after harvest by mushroom growers (White Prince, NSW, Australia) were purchased by The Shoreline for inclusion in RC participant meals. Participants in IL were provided with punnets of vitamin D mushrooms provided by the Australian Mushroom Growers Association to include in meals at any time of the day. As a commercially available mushrooms, the estimated nutrient composition of raw and cooked vitamin D mushrooms (*Agaricus bisporus*) is included in the Australian Food Composition Database (AFCD) (36). Ethical approval was obtained from the Bellberry Human Research Ethics Committee (Application No. 2023-10-1237). All participants gave verbal and written informed consent and could decline the invitation to participate in the study at any time without disadvantage.

### 2.2 Recruitment

As a feasibility study, a formal sample size calculation was not performed and as many eligible participants as possible were recruited to explore the practical aspects of implementation, consistent with CONSORT guidelines (43) and other feasibility studies (44, 45). Participants were recruited from RC and IL through posters at the facility, through media (newspaper articles and Facebook posts), via electronic direct mail and a formal in-person information session held by the study nurse and educator (LD) and principal investigator (FFM). Staff participated in the qualitative data collection, including outcome surveys and focus groups. Participants were included if they could speak and read English, and were willing to order (RC) or make (IL) at least one vitamin D mushroom meal per day for 4 weeks. Participants were excluded if unable or unwilling to provide informed consent or unable to consume mushrooms (e.g., fungi allergy). On enrolment, self-reported data were collected on demographics and general health.

### 2.3 Mushrooms on the menu

#### 2.3.1 Residential care

During baseline, participants in RC consumed meals from the standard food service menu (Supplementary Table 1). Breakfast was served buffet-style, with recipes varying depending on the availability of ingredients and portion sizes dependent on participant self-service; therefore, breakfasts were excluded from the RC intervention and data collection. Data from lunches and dinners were collected over the four-week periods of each study phase. The MOM menu was designed to maintain the variety of meals in the standard menu but provide at least one vitamin D mushroom meal option at lunch and dinner daily. Each vitamin D mushroom meal contained the equivalent of a serve of vegetables (75 g) using vitamin D mushrooms, delivering approximately 18 μg of vitamin D (36). The food service manager adapted the standard menu, and the internal consultant dietitian reviewed the menu for nutritional adequacy. A total of 123 meal options were provided for lunch and dinner in both the standard menu and MOM. A total of 28 meals contained mushrooms. Twenty-six of the standard meals were modified to include vitamin D mushrooms, plus two newly created mushroom meals replaced two of the standard menu meals (Supplementary Table 1). For the 26 modified standard meals, up to 20% of the total weight was replaced with vitamin D mushrooms to avoid changes to the original flavour profiles. During MOM, participants in RC were instructed to choose at least one mushroom meal daily for 4 weeks however compliance was voluntary.

#### 2.3.2 Independent living

During baseline, participants in IL were asked to continue their usual diet, including preparing and cooking meals. During MOM, participants in IL were given 600 g (2 × 300 g punnets) of fresh vitamin D mushrooms weekly to use and instructed to make at least one mushroom meal per day for 4 weeks. They were provided with a MOM study recipe book for meal inspiration and to assist with making recipes that provide at least 75 g of vitamin D mushrooms per serve.

### 2.4 Data collection and analysis

#### 2.4.1 Dietary data

Dietary data for the participants in RC for at least four lunch and four dinner meals were collected at randomized times during the standard menu and MOM phases. To determine actual intake and minimize participant burden, research assistants attended each meal service in RC and manually recorded the meal name and plate waste using a seven-point visual rating scale (100, 90, 75, 50, 25, 10 and 0% of the meal left) (46).

Dietary data for participants in IL were collected with the web-based Automated Self-Administered 24-h Dietary Assessment Tool (ASA24) used in research and clinical settings to assess intake (47). The ASA24 prompts users to recall all foods and beverages consumed in the previous 24 h, including questions on portion sizes, preparation methods, and added ingredients. The mean intake of at least two 24-h recalls per participant collected at randomised occasions was calculated during each phase (standard menu and MOM).

Recipes from the standard menu and MOM were added to FoodWorks v10.0 (Xyris Software Pty Ltd., Australia) (48), from which the nutritional composition of each menu was determined. Food composition data for both FoodWorks and ASA24 were derived from the food composition database from Food Standards Australia New Zealand (FSANZ) (36). The mean energy, macronutrient, total vitamin D, vitamin D_2,_ and sodium intakes were calculated by meals for each study phase to reduce bias from varying number data collection points from each participant depending on how many meals they reported. The total nutrient provision and intake for lunches and dinners and the sum of lunches and dinners were calculated for participants in RC. For participants in IL, daily mean intakes were calculated. Nutrient intake data were obtained from dietary intake only and did not include vitamin D from supplementation.

#### 2.4.2 Outcome survey

An outcome survey for each RC, IL, and staff participant was used to collect their experiences during the MOM. The RC & IL surveys were completed after meals by researchers and self-administration respectively, and staff surveys were completed at study end. Among participants in RC, Likert scale ratings (very poor, poor, average, good, excellent) were collected on the taste, presentation, and overall enjoyment of vitamin D mushroom meals and the likeliness to re-order the meal (yes, no, unsure). For participants in IL, data for the same Likert scale questions were collected with self-administered online surveys through QuestionPro survey software (Austin, Texas, USA), along with a rating on the texture of the mushroom meal (excellent, good, average or below), the likelihood that the meal would be cooked again (yes/no) and if any side effects were noticed when eating the mushroom meals (yes/no).

Staff insights on preparation, storage, and knowledge of health benefits of vitamin D mushrooms, and experiences with and future adoption of a vitamin D mushroom menu were gathered using a 6-point Likert scale (strongly disagree, disagree, neither agree nor disagree, agree, strongly agree, not applicable) through QuestionPro. When questions did not apply to a participant’s role in the facility, they could select ‘not applicable’.

For 2 weeks post-MOM, an outcome survey was administered to staff through QuestionPro. Staff were asked about their area of work at the facility (companion, housekeeping, food service, kitchen or other), and using a 5-point Likert scale, if the vitamin D mushrooms were easy to prepare, if they had adequate equipment, training, cold storage, time to prepare and support to include the mushroom meal on the menu, if they encouraged residents to order the mushroom meals, if they understand the health benefits of vitamin D mushrooms, if their mushroom consumption has increased, and if eating vitamin D mushrooms is a suitable alternative to supplementation (strongly agree, agree, neither agree nor disagree, disagree or strongly disagree, not applicable). When questions did not apply to a participant’s role in the facility, they could select ‘not applicable’.

#### 2.4.3 Focus groups

Focus group interviews were conducted by a research nurse and educator (LD) with technical support from a research assistant after the intervention phase. The sessions were audio-recorded and guided by a semi-structured focus group format developed for the MOM study. The Shoreline staff, and participants in RC and IL voluntarily attended different focus group sessions that captured data relevant to each group’s experiences and study methods (Supplementary Appendix 1). All audio-recorded data were transcribed, and data were imported into NVivo (Version 14, Lumivero Pty Ltd) (49) for coding and data management. The data were analysed by two researchers (ED, MB) using a thematic approach reflecting the process proposed by Braun and Clarke (50). This included familiarization by reading, identifying meaning and patterns in the data, generating initial codes, generating and reviewing themes, then defining and naming themes to finalize the analysis (51). This approach was appropriate for the study’s aims, which were not to build a theory or interpret the meaning of an experience but to stay close to the data and the themes that emerged from different participant perspectives (52).

#### 2.4.4 Cost data

Cost data for standard meals and those adapted to include vitamin D mushrooms were provided by the chef at the residential aged care facility. The total cost in AUD of the 26 standard menu recipes before and after their adaptation to include 75 g of vitamin D mushrooms and the cost of replacing two standard menu meals with two new vitamin D mushroom meals were calculated.

#### 2.4.5 Statistical analyses

Primary outcome measures included a change in the nutritional provision of the MOM compared to the standard menu for residents in RC, total vitamin D intakes, the contribution of the vitamin D from the vitamin D mushrooms (D_2_) to total vitamin D intake, and the proportion of participants meeting the AI set out in the Australian Nutrient Reference Values (NRVs) for vitamin D. Among participants in RC, vitamin D intake was calculated from lunch and dinner separately and as the total contribution from lunches and dinners combined. In contrast, levels among participants in IL were calculated based on their daily intake as vitamin D mushrooms could be included in meals throughout the day. For participants in RC and IL the change in energy, macronutrient, sodium and other micronutrient intakes were measured during MOM compared to the standard menu phase.

Secondary outcome measures included experiences around the acceptability of the vitamin D mushroom meals, impact on own behaviors, experiences around health and wellbeing, and learnings around the feasibility of implementation. For staff, secondary outcomes included their experiences on storage, preparation, health benefits and continued use of vitamin D mushrooms.

Changes in the estimated nutritional provision between the standard menu and MOM were determined using independent t-tests, and when statistically significant, a percentage increase or decrease compared to baseline was reported. The mean cost of vitamin D mushroom meals (n = 26) was calculated and differences with the cost of their recipe in the standard menu described.

Changes in estimated total mean nutrient intakes between study phases were compared using t-tests and when statistically significant, the percentage increase or decrease was recorded. The estimated percentage contribution of vitamin D_2_ from vitamin D mushrooms to total vitamin D intakes for RC (lunches and dinners) and IL (daily) were calculated for each study phase. NRV’s were used to determine the number of participants meeting vitamin D targets. The AI for adults aged 51–70 years is 10 μg/day, and for those aged over 70 years is 15 μg/day (53).

Statistical analyses were performed using Excel and JMP Statistical Software (Pro V.14.2.0; SAS Institute Inc., Cary, NC, United States 27513). A *p*-value threshold of <0.05 was considered statistically significant.

## 3 Results

### 3.1 Participant characteristics

#### 3.1.1 Residential care

Seventy-five residents of RC enrolled in the study, 15 were withdrawn due to hospitalization or poor health (*n* = 11), loss of life (*n* = 3), and personal choice (*n* = 1,) with 60 completing the study. A voluntary focus group was conducted with a subset of 10 participants who expressed interest in taking part. The mean age (±SD) of participants in RC was 87.2 (±6.5) y, BMI was 26.4 (±4.5) kg/m^2^, most were female (72%), and most were in good to excellent health (75%; Table 1).

**Table 1:** Baseline characteristics of The Shoreline residential aged care facility study participants.

Characteristic	Residential care (*n* = 60)	Independent living (*n* = 12)	Staff (*n* = 12)
	Mean (SD)	Mean (SD)	Mean (SD)
Age (y)	87.2 (6.5)	72.6 (4.3)	37.4 (15.3)
Height (m)	1.6 (0.1)	1.6 (0.1)	1.7 (0.1)
Weight (kg)	68.4 (15.2)	76.7 (11.8)	70.1 (15.0)
BMI (kg/m^2^)	26.4 (4.5)	29.5 (6.4)	25.1 (6.3)
	n (%)	n (%)	n (%)
Sex			
Female	43 (72.0)	9 (75.0)	8 (66.7)
Male	17 (28.0)	3 (25.0)	4 (33.3)
Health status			
Poor to fair health	15 (25.0)	0.0 (0.0)	1 (8.3)
Good to excellent health	55 (75.0)	12 (100)	11 (91.7)
Mushroom consumption frequency			
≥ once per week	27 (45.0)	9 (75.0)	5 (41.7)
< once per week	34 (55.0)	3 (25.0)	7 (58.3)

BMI, Body Mass Index. The sex variable should be on a different line to *n* (%).

#### 3.1.2 Independent living

Twelve residents of IL were enrolled and completed the study, and a subset of six participated in the focus group and 11 had complete data. The mean age of participants in IL was 71.6 (±4.3) y, and BMI was 29.5 (±6.4) kg/m^2^. Three-quarters (75%; *n* = 9) were female and reported consuming mushrooms at least once per week at baseline, while all participants were in good to excellent health (Table 1).

#### 3.1.3 Staff

Twelve staff completed the outcome survey, and four participated in the focus group. The mean age of staff that completed the outcome surveys and focus groups was 37.4 (±15.3) y, and the mean BMI was 25.1 (±6.3) kg/m^2^. Two-thirds were female (66.7%), and most were in good to excellent health (91.7%) (Table 1).

### 3.2 Menu nutrient provision

The mean total vitamin D provided by the lunches and dinners during MOM was 180% higher than the standard menu (7.0 μg vs. 2.5 μg, *p* < 0.0001; Table 2). No differences were found between the menus for energy, macronutrients, or any other micronutrient (Table 2).

**Table 2:** Nutrition provision by the RC standard menu (baseline) and mushrooms on the menu (MOM).

Nutrient	Standard Menu		MOM		t-test
	Mean provision per meal (SD)	CI	Mean provision per meal (SD)	CI	*p* (2-tailed)
Energy (kJ)	1910 (714.7)	1782–2037	1924 (702.9)	1798–2049	0.9
Protein (g)	27.2 (13.5)	24.7–29.6	27.6 (13.4)	25.2–30.0	0.8
Carbohydrate (g)	38.3 (21.4)	34.4–42.1	38.0 (21.1)	34.2–41.8	0.9
Fibre (g)	10.1 (8.8)	8.6–11.7	10.4 (8.8)	8.8–12.0	0.9
Total fat (g)	19.1 (10.5)	17.2–21.0	19.4 (10.6)	17.5–21.2	0.9
Saturated fat (g)	6.4 (4.7)	5.6–7.3	6.6 (4.8)	5.8–7.5	0.8
Vitamin A eq. (μg)	777.6 (626.1)	665.8–889.3	805.2 (657.5)	687.9–922.6	0.7
Vitamin C (mg)	161.7 (137.9)	137.1–186.4	166.3 (137.5)	141.7–190.8	0.8
Vitamin D, total (μg)	2.5 (3.1)	1.9–3.0	7.0 (9.1)	5.3–8.6	**<0.0001**
Vitamin D_2_ (μg)	0.1 (0.4)	0.1–0.2	4.6 (8.2)	3.1–6.0	**<0.0001**
Vitamin E (mg)	5.5 (3.6)	4.8–6.1	5.5 (3.5)	4.9–6.1	1.0
Thiamine (mg)	0.3 (0.3)	0.3–0.4	0.3 (0.2)	0.3–0.7	0.9
Riboflavin (mg)	0.3 (0.2)	0.3–0.4	0.3 (0.2)	0.3–0.4	1.0
Niacin eq. (mg)	11.9 (7.3)	10.6–13.2	12.4 (7.3)	11.1–13.8	0.6
Vitamin B6 (mg)	1.0 (0.9)	0.9–1.2	1.0 (0.9)	0.9–1.2	1.0
Folate DFE (μg)	136.2 (64.8)	124.7–147.8	140.6 (65.5)	128.9–152.3	0.6
Vitamin B12 (μg)	0.9 (0.9)	0.8–1.1	0.9 (1.0)	0.8–1.1	0.8
Calcium (mg)	171.5 (111.3)	151.6–191.4	174.5 (113.9)	154.2–194.8	0.8
Iodine (μg)	19.3 (29.7)	14.0–24.6	18.9 (29.4)	13.7–24.2	0.9
Iron (mg)	3.8 (1.9)	3.4–4.1	3.8 (1.9)	3.5–4.2	0.8
Magnesium (mg)	88.8 (32.7)	83.0–95.0	90.5 (32.7)	84.7–96.3	0.7
Phosphorous (mg)	381.5 (155.7)	353.7–409.3	397.4 (163.6)	368.2–426.6	0.4
Potassium (mg)	1157.8 (538.9)	1061.6–1,254	1207.5 (557.7)	1,108–1,307	0.5
Selenium (μg)	23.7 (18.6)	20.4–27.0	26.1 (18.3)	22.8–29.3	0.3
Sodium (mg)	713.8 (401.4)	642.2–785.5	683.6 (380.9)	615.6–751.6	0.6
Zinc (mg)	3.2 (2.1)	2.8–3.6	3.2 (2.1)	2.8–3.6	1.0

Total number of meals per menu: Standard Menu = 123, MOM = 123 (28 meals included vitamin D mushrooms; 95 standard menu meals); SD, standard deviation; CI, confidence interval; eq., equivalents; DFE, dietary folate equivalents. Statistically significant values highlighted in bold.

### 3.3 Nutrient intakes

#### 3.3.1 Residential care

The mean proportion of each meal consumed by participants was 80%, which did not differ between the standard and MOM menus (Supplementary Table 2).

Relative to the standard menu, there were no differences in the provision of energy, protein, total fat, or sodium intakes provided during the MOM. In contrast, saturated fat intake was slightly higher during the MOM than the standard menu (Table 3). Across lunches only, carbohydrate provision was lower (26.8 vs. 24.9 g, *p* = 0.03), and fibre higher (6.9 vs. 7.6 g, *p* = 0.04) during the MOM compared to the standard menu (Table 3). The complete nutrient data can be found in Supplementary Table 3.

**Table 3:** Energy, macronutrient, vitamin D and sodium intakes by meals among RC participants during the standard menu and MOM.

Nutrient	Standard menu	MOM			*t*-test	% change
	Mean (SD)	CI	Mean (SD)	CI	*p* (2-tailed)	
Lunches						
Energy (kJ)	1,674 (424)	(1564–1783)	1,694 (486)	(1569–1820)	0.8	
Protein (g)	26.9 (8.1)	(24.8–29.0)	26.7 (7.5)	(24.7–28.6)	0.9	
Carbohydrate (g)	26.8 (7.1)	(25.0–28.7)	24.9 (7.4)	(23.0–26.9)	**0.03**	−7.1
Fibre (g)	6.9 (2.0)	(6.3–7.4)	7.6 (2.6)	(6.9–8.3)	**0.04**	10.2
Total fat (g)	19.0 (6.2)	(17.4–20.6)	19.9 (6.9)	(18.1–21.7)	0.3	
Saturated fat (g)	5.9 (2.2)	(5.4–6.5)	6.9 (2.4)	(6.3–7.5)	**0.004**	17.0
Total vitamin D (μg)	2.7 (1.5)	(1.4–3.1)	8.6 (5.5)	(7.2–10.0)	**<0.0001**	219
Vitamin D_2_ (μg)	0.2 (0.8)	(0.03–0.5)	6.1 (4.9)	(4.8–7.4)	**<0.0001**	2,950
Sodium (mg)	664 (276)	(593–736)	641 (247)	(577–705)	0.5	
Dinners						
Energy (kJ)	1,610 (477)	(1487–1733)	1,571 (506)	(1440–1702)	0.5	
Protein (g)	24.6 (8.7)	(22.3–26.8)	24.9 (8.8)	(22.7–27.2)	0.7	
Carbohydrate (g)	32.1 (10.3)	(29.4–34.7)	31.3 (10.0)	(28.7–33.9)	0.6	
Fibre (g)	8.0 (4.9)	(6.7–9.2)	8.2 (3.2)	(7.4–9.1)	0.7	
Total fat (g)	15.7 (5.4)	(14.3–17.1)	14.7 (5.5)	(13.3–16.1)	0.1	
Saturated fat (g)	5.1 (2.1)	(4.6–5.7)	5.7 (2.3)	(5.2–6.3)	**0.04**	11.8
Total vitamin D (μg)	3.2 (1.7)	(2.8–2.7)	10.1 (5.2)	(8.7–11.4)	**<0.0001**	216
Vitamin D_2_ (μg)	0.2 (0.2)	(0.2–0.3)	7.4 (4.5)	(6.2–8.6)	**<0.0001**	3,600
Sodium (mg)	584 (195)	(533–634)	545 (211)	(491–600)	0.2	
Totals lunches and dinners						
Energy (kJ)	3,284 (682)	3,107–3,460	3,265 (763)	3,068–3,462	0.8	
Protein (g)	51.2 (12.6)	48.0–54.5	51.8 (12.9)	48.5–55.1	0.7	
Carbohydrate (g)	58.9 (13.2)	55.5–62.3	56.2 (13.1)	52.8–60.0	0.09	
Fibre (g)	14.8 (5.4)	13.5–16.2	15.8 (4.3)	14.7–16.9	0.2	
Total fat (g)	34.7 (8.3)	32.6–36.9	34.6 (9.7)	32.1–37.1	0.9	
Saturated fat (g)	11.1 (3.2)	10.3–11.9	12.7 (3.7)	11.7–13.6	**0.0004**	14.4
Total vitamin D (μg)	6.0 (2.4)	5.4–6.6	18.7 (6.7)	17.0–20.4	**<0.0001**	212
Vitamin D_2_ (μg)	0.5 (0.8)	0.3–0.7	13.5 (5.8)	12.0–15.0	**<0.0001**	2,600
Sodium (mg)	1,248 (362)	1,154–1,342	1,187 (351)	1,096–1,278	0.2	

Participants *n* = 60; MOM, mushrooms on the menu; SD, standard deviation; CI, confidence intervals. Statistically significant values highlighted in bold.

**Figure 2:**
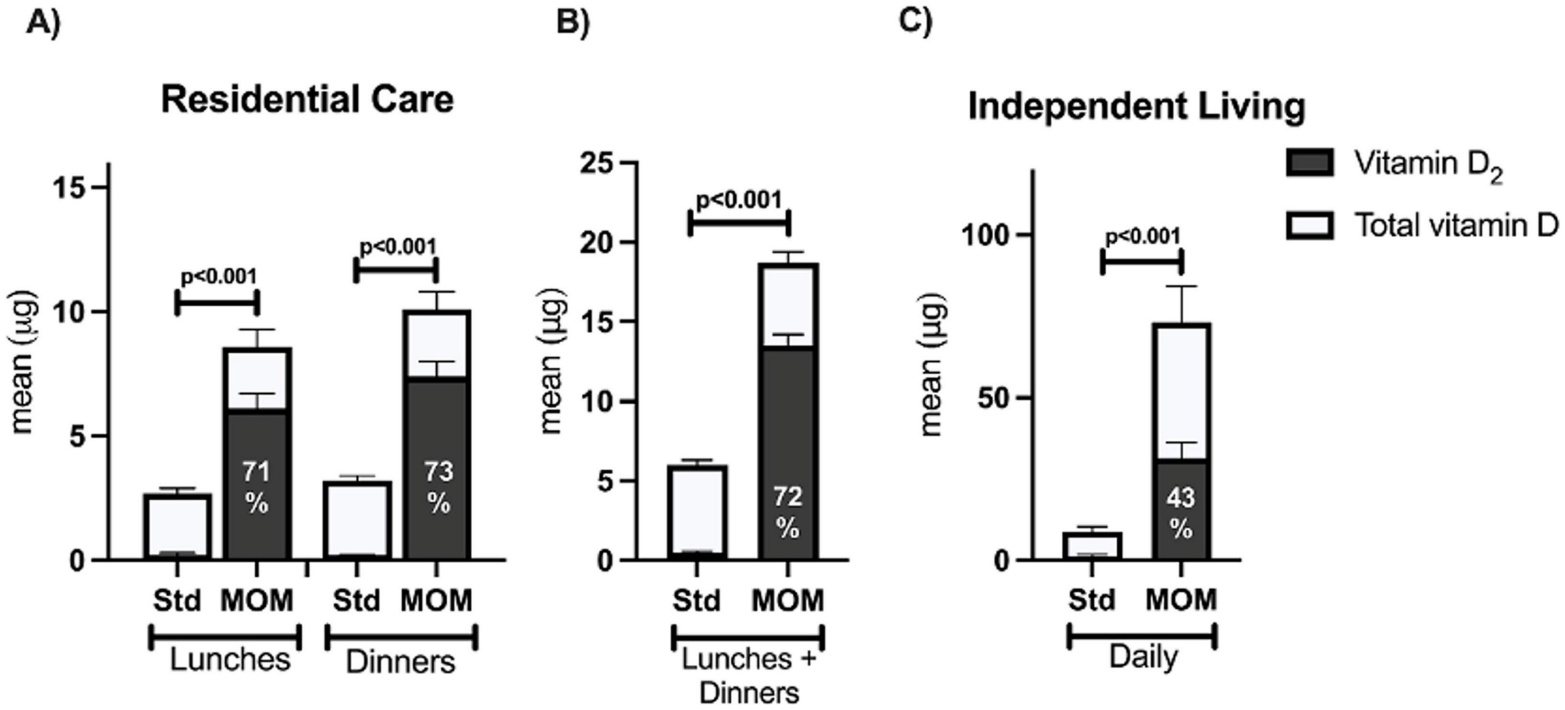
The percentage contribution of vitamin D_2_ to total vitamin D intakes during MOM. RC participants *n* = 60; IL participants *n* = 11; Std, standard menu; MOM, mushrooms on the menu. The percentage contribution of D_2_ to total vitamin D intakes during MOM from **(A)** lunches and dinners among RC residents, **(B)** Lunches and dinners combined among RC residents, **(C)** Across the day among IL residents. *The percentage contribution of vitamin D_2_ to total vitamin D intakes during the baseline phase: RC lunches 7.2%, RC dinners 6.3%, RC lunches + dinners 8.3%, IL daily 14.9%.

Mean total vitamin D intake was three times higher during the MOM compared to the standard menu (sum of lunches and dinners 18.7 vs. 6.0 μg, *p* < 0.0001; Table 3). Specifically, vitamin D_2_ was substantially higher and contributed more than 70% of the total vitamin D intake during MOM compared to the standard menu (Figure 2).

**Figure 3:**
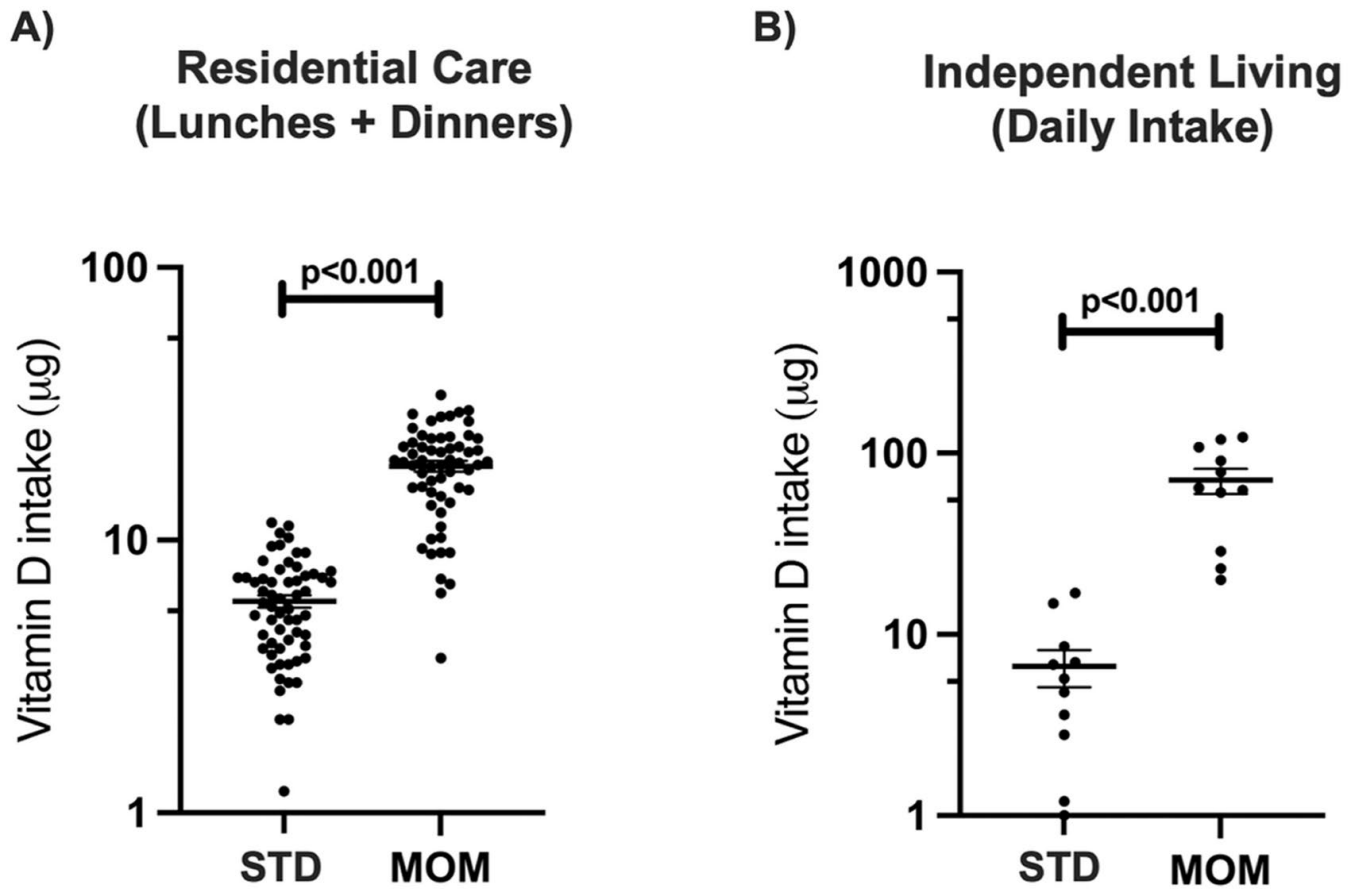
Number of participants who met the Adequate Intake* for vitamin D. RC participants *n* = 60; IL participants *n* = 11; S, standard menu; MOM, mushrooms on the menu. * AI; Adequate intake is 10 μg/day for 51–70 year olds and 15 μg/day for >70y.

The proportion of participants meeting the AI for vitamin D using nutrient intake data from only their lunches and dinners increased from 0% during the standard menu to 75% during the MOM (Figure 3). The mean vitamin D intake from lunches and dinners during the MOM (18.7 μg) represents 125% of the AI for over 70 years (15 μg/day).

#### 3.3.2 Independent living

Mean daily total vitamin D and vitamin D_2_ intakes were higher during MOM compared to the standard menu (73.1 vs. 8.7 μg and 31.1 vs. 1.3 μg respectively, *p* < 0.0001 for both), while no differences in intakes were found for energy, macronutrients and sodium (Table 4). The contribution of vitamin D_2_ to total vitamin D intake was higher during MOM than the standard menu phase (42.8% vs. 24.6%, *p* < 0.0001; Figure 2), with no statistically significant increase in vitamin D_3_.

**Table 4:** Mean daily energy, macronutrient, vitamin D and sodium intakes among IL participants during the standard menu and MOM.

Nutrient*	Standard menu	MOM			*t*-test	% change
	Mean (SD)	CI	Mean (SD)	CI	*p* (2-tailed)	
Energy (kJ)	8,822 (3063)	6,764–10,880	8,192 (2444)	6,550–9,834	0.6	
Protein (g)	91.2 (32.2)	69.6–113	88.4 (19.5)	75.3–101.5	0.8	
Carbohydrate (g)	185 (69.5)	138.3–234	168.9 (74.8)	119–219	0.6	
Fibre (g)	25.1 (9.1)	19.1–31.2	24.0 (8.0)	18.7–29.4	0.8	
Total fat (g)	90.6 (28.0)	71.8–109	87.5 (26.0)	70.1–105	0.8	
Saturated fat (g)	34.1 (11.7)	26.2–41.9	32.2 (12.4)	23.9–40.6	0.7	
Total vitamin D (μg)	8.7 (7.6)	3.6–13.8	73.1 (34.2)	50.1–96.1	**<0.0001**	740
Vitamin D_2_ (μg)	1.3 (1.7)	0.1–2.4	31.3 (16.6)	20.2–42.5	**<0.0001**	2,309
Sodium (mg)	2073 (834)	1,512–2,633	1893 (907)	1,284–2,502	0.6	

Participants *n* = 11; MOM, mushrooms on the menu; SD, standard deviation; CI, confidence intervals. *Based on at least two 24-h recalls per participant. Statistically significant values highlighted in bold.

The proportion of participants meeting the AI for vitamin D increased from 8.3% during the standard menu to 100% during the MOM (Figure 3). Participants in IL consumed more than one 75 g serve of vitamin D mushrooms daily, with a mean vitamin D intake of 73.7 μg during the MOM, and well above the AI for over 70 years (15 μg/day).

### 3.4 Qualitative data on feasibility

#### 3.4.1 Outcome surveys

Participants in RC rated the vitamin D mushroom meals as good or excellent for taste (78%), presentation (75%), and overall enjoyment (78%), and 66.1% would order the meals again (Supplementary Table 4). 100% of participants in IL rated the vitamin D mushroom meals as good to excellent for taste, presentation, texture and overall enjoyment, said they would cook the meal again and reported no side effects when eating the vitamin D mushrooms (Supplementary Table 4).

More than 75% of surveyed staff agreed that vitamin D mushroom meals were easy to prepare, that adequate preparation equipment, time and storage were available, that they were adequately trained to prepare the meals, that they had sufficient administrative support to include the vitamin D mushroom meals, that they encouraged residents to order vitamin D mushroom meals, that they understood their health benefits, that they support their inclusion in aged care food service menus and that the MOM study increased their intake of mushrooms (Supplementary Table 5).

#### 3.4.2 Focus groups

Three major themes were identified from the focus group data (Table 5). The first theme was understanding participants’ perception of the vitamin D mushroom menu’s acceptability and enjoyment. Participants in RC found vitamin D mushrooms enjoyable when meals were varied to include mushrooms as both a hero and complementary ingredient. Acceptability was high when vitamin D mushrooms were consumed at lunch and dinner, when not consumed every day as a hero ingredient, or when unaware consumption was occurring daily. Participants in IL consistently reported very positive experiences with the vitamin D mushroom meals, likely due to their greater independence and ability to incorporate vitamin D mushrooms into their diet compared to those in RC, including cooking the meals themselves, selecting recipes that they enjoy, and deciding when and how to have the vitamin D mushrooms. Practical considerations related to meal variety, eating occasion frequency and preparation preferences to increase acceptability and enjoyment of vitamin D mushroom meals are summarized in Supplementary Table 6.

**Table 5:** Themes identified in qualitative analysis of focus group data (RC, IL, and staff).

Themes	Description
Mushrooms are enjoyable when there is adequate variety of meals, and when eating occasion frequency and preparation preferences are taken into account.	Residents enjoyed the mushrooms meals, but they wanted variety in the way they consume mushrooms and prefer a combination of meals that showcase mushrooms, and others where mushrooms are used as a complimentary ingredient. Ongoing daily consumption of meals that heavily showcase mushrooms was not preferred. The feedback from independent living residents was positive. Their higher level of independence compared to residents afforded them greater choice about how they incorporated mushrooms into their diet during the study (i.e., cooking themselves, with s of their choosing, to the frequency they wanted). This emphasises the importance of variety, eating occasion frequency and preparation preferences when it comes to enjoying regularly consuming mushrooms.
Both staff and residents gained knowledge on mushrooms and their health benefits.	Staff and residents alike expressed the key impact of taking part in the trial was their increased knowledge, in particular around the method to UV-expose mushrooms and their ability to provide dietary vitamin D. Some independent living residents reported feeling very positive about their learnings, and how this had impacted their behavior with regards to preparing and eating mushrooms.
Ensuring residents order and eat mushroom meals is challenging but could be overcome with strategic menu planning.	Coordinating the mushroom orders for all residents was challenging for staff. In part, it was an additional task to their usual responsibilities, and it required close monitoring as changes to residents’ meal orders changed often and at short notice. To feasibly implement a menu containing UV-exposed mushrooms, strategic menu planning, alongside implementation processes with low staff burden, could overcome these barriers.

The second theme related to understanding the impact on participant knowledge of mushrooms and their health benefits (Table 5). New knowledge about mushroom preparation, cooking practices and the health benefits of consuming vitamin D mushrooms led participants in IL to intend to continue consuming mushrooms, and RC staff to intend to provide vitamin D mushrooms on the menu after the study ended. RC participants reported an understanding of the method to UV-expose mushrooms and their ability to provide vitamin D from education sessions. Further considerations to understand the impact of the MOM study on participant knowledge on mushrooms are presented in Supplementary Table 6.

The third theme related to the feasibility of implementing a vitamin D mushroom menu in an aged care facility (Table 5). Clinical and food service staff expressed the need for low-burden processes as there were additional responsibilities related to the study (e.g., tracking vitamin D mushroom meal ordering and consumption) that were difficult for staff to accommodate in a demanding aged care environment. Practical challenges to successful implementation were overcome with strategic menu planning, where challenges identified during recipe development related to balancing meal flavors while maintaining acceptable meal portion sizes, forecasting quantity and cost of raw ingredients, and keeping staff burden to implement low. Further considerations for strategic menu planning are presented in Supplementary Table 6. No adverse effects were reported in either group.

#### 3.4.3 Cost

Vitamin D mushrooms were purchased for the RC menu at $14.00 AUD a kilogram, costing $1.05 per 75 g serving. The mean difference in cost between the mushroom meals and the standard meals they replaced was $0.55 AUD (Supplementary Table 7). The average cost of the 26 standard meals was $3.35, and when mushrooms were added, the average cost was $3.85 AUD, while four mushroom meals were cost-neutral (Supplementary Table 7). In the focus group, the facility chef advised that adding mushrooms to the menu did not impact the total food service budget and that he could deliver the menu with the additional cost absorbed through recipe modifications and cost savings on having less expensive ingredients in some recipes.

## 4 Discussion

This was the first study to evaluate the feasibility and impact on vitamin D menu provision and intake with vitamin D mushrooms in a residential aged care facility. The results indicate that adapting recipes on a RC food service menu to include at least one meal option at both lunch and dinner that contained 75 g of vitamin D mushrooms per serve and encouraging vitamin D mushroom consumption by those in IL can substantially increase the provision and intake of vitamin D. Adding mushroom meals were a feasible, well-liked and accepted method to increase vitamin D intake that did not compromise the nutritional value of the menu offered, and a desire to continue with the MOM was evident. Strategic recipe development was essential to reduce staff burden, provide menu variety that catered to resident preferences, and reduce the impact on existing food service budgets. Including commercially available vitamin D mushrooms that are UV-treated after harvest removed the need for staff to manually raise vitamin D content of the mushrooms through sun exposure. Education on preparation practices and the health benefits of vitamin D mushrooms were also required for successful implementation. The feasibility of incorporating vitamin D mushrooms into residential aged care menus was demonstrated and provides a foundation for future studies evaluating long-term changes in participants’ biological vitamin D levels following similar integration of UV-treated mushrooms into institutional and independent living settings.

To our knowledge, this is the first time a food-based intervention has been shown to address low vitamin D intake using mushrooms in an aged care setting. Findings demonstrate that aged care facilities can provide substantial dietary vitamin D that exceeds daily targets by including vitamin D mushrooms on a food service menu (RC) and in resident own meal preparation (IL). Enhancing the lunch and dinner menus daily with vitamin D mushrooms increased the total vitamin D provided in RC by 180%, tripling vitamin D intake for this group compared to the standard menu. Given a large proportion of RC participants had intake above the AI while excluding the contribution from breakfast, residents’ vitamin D intake may be even higher than reported. Findings are supported by Australian modelling data that reported one serve per day (75 g) of vitamin D mushrooms can help all Australians meet their vitamin D targets, including those aged 70 years and older (33). Nationally representative data from the Netherlands (24) found that substituting 60–84 g of regular mushrooms for vitamin D mushrooms increased dietary vitamin D intake, while an 84 g serving of vitamin D mushrooms in a population-based US sample almost doubled vitamin D intake without impacting energy, sodium or fat (54). Similarly, an increase in vitamin D intake was evident during MOM without impacting energy intake. This is important given natural dietary sources of vitamin D from animals are often high in energy, which could introduce additional calories to a food service menu. Furthermore, one serve of vitamin D mushrooms can provide a natural, vegan source of vitamin D that can meet dietary targets (33) and provides a strategy that aligns with dietary guidelines globally that are shifting away from animal foods (55, 56).

Findings support previous work indicating that consumption of vitamin D mushrooms can provide a wider variety of nutrients than supplementation (24). In addition to vitamin D, mushrooms are a source of micronutrients including niacin, folate, selenium, phosphorous, and potassium (36). In the current study, intakes of these nutrients (except selenium) by participants in RC increased with consumption of vitamin D mushrooms, which aligns with findings from dietary modeling (24). Previous research, including a systematic review with meta-analysis has reported that vitamin D mushrooms effectively increase vitamin D_2_ status when fresh (34, 35, 57–60) or dried (61–64), although results may vary with baseline vitamin D status. While supplementation can effectively address low vitamin D status, it is often poorly implemented (10, 11) and included at an additional cost to food (65) in aged care. Foods offer diverse (66), bioavailable and easily absorbed nutrients (67) with various health benefits (66) that have the potential to reduce the healthcare and quality of life costs associated with vitamin D deficiency, such as the risk of falls and fractures (1, 3). Further research investigating vitamin D status in aged care following a food-based intervention and the impact on healthcare cost and utilization is warranted, along with a comparison to supplementation.

Results indicate potential for broader incorporation as a staple ingredient in residential aged care facility menus from high ratings for taste, presentation, and overall enjoyment of the mushroom meals among participants and a high level of acceptance and satisfaction. The high acceptability of mushrooms as both a feature or complimentary ingredient demonstrates their versatility, while staff intention to continue to offer vitamin D mushroom meals, and resident interest to consume these meals upon trial completion, showcase the effectiveness of the intervention to change knowledge and behavior beyond the study completion. Similar to other studies in residential aged care facilities, these findings highlight the need for culinary flexibility and responsiveness to residents’ tastes when designing meals with vitamin D mushrooms (68–70) and demonstrate the importance of education and training on health benefits and preparation methods (71, 72) for successful adoption. A phased approach starting with pilot programs before broader implementation is recommended to help identify and address potential issues, optimize menu design and recipe development, and minimize staff burden. The successful integration of vitamin D mushrooms into aged care facility menus can be supported through participant and staff education that covers the health benefits of vitamin D mushrooms, preparation and cooking techniques, and recipe designs that maintain the flavor profiles of existing menus. Replication of this research in other aged care facilities is required to optimize intervention administration, vitamin D intake and health in this high-risk population group.

Strengths of this study include the breadth of residents (RC and IL), the inclusion of resident and staff education sessions, and the mixed methods design that incorporates both numerical data and experiences and perspectives. Further, the amount of vitamin D mushrooms added to the menu proved feasible, and was easily consumed and incorporated into meals in line with a serving of vegetables in the Australian Dietary Guidelines. Limitations include the small sample size for participants in IL, the voluntary nature of daily mushroom meal consumption which may have limited assessment of the interventions full effect, and the use of estimated vitamin D content from the AFCD (36) rather than direct analysis. However, this database is a nationally recognized resource for food nutrient levels and existing evidence indicates that vitamin D levels in mushrooms remain relatively stable after cooking (27, 73). Even though breakfast was excluded from the analysis due to the feasibility of measuring dietary intake at the buffet occasion, it is well reported that lunches and dinners typically contribute the most to daily nutrient intake than other eating occasions (6, 74). Among those in RC, comparisons against the AI were reported as the proportion of the AI achieved to account for this limitation. It is also well documented that portion consumption estimates and self-reported dietary data through 24-h recall can introduce bias (75), therefore, repeated recalls were used to account for some of the errors in reporting. Focus groups may be subject to social desirability bias where participants provide expected responses rather than true experiences and may miss differing experiences from non-volunteers (76). However, feasibility studies focus on assessing processes, testing interventions and incorporating qualitative data to refine future implementation (77). Finally, a mushroom-based food intervention to increase vitamin D intake will not be applicable to those who do not enjoy eating mushrooms, as this study specifically recruited residents who were willing to consume mushrooms daily.

## 5 Conclusion

The MOM study provides valuable insights into the feasibility, acceptability, and ability of vitamin D mushrooms to improve vitamin D intake and assist aged care residents meet their recommended targets. Findings demonstrate that a food service menu with at least one meal option at both lunch and dinner that contains 75 g of vitamin D mushrooms is a feasible food-first approach to increase the vitamin D provision and intake of residents, with the potential for minimal impact on existing food service budgets with strategic recipe development. Given the widespread prevalence of vitamin D deficiency in aged care, further research is required to replicate this work both directly and conceptually across other aged care facilities to optimize health in this high-risk population group.

## Data Availability

The raw data supporting the conclusions of this article will be made available by the authors, without undue reservation.

## References

[1] Meehan M Penckofer S. The role of vitamin D in the aging adult. J Aging Gerontol. (2014) 2:60–71. doi: 10.12974/2309-6128.2014.02.02.1, PMID: 25893188 PMC4399494

[2] Feehan O Magee PJ Pourshahidi LK Armstrong DJ McSorley EM. Vitamin D deficiency in nursing home residents: a systematic review. Nutr Rev. (2022) 81:804–22. doi: 10.1093/nutrit/nuac091, PMID: 36367832 PMC10251303

[3] Timpini A Pini L Tantucci C Cossi S Grassi V. Vitamin D and health status in elderly. Intern Emerg Med. (2011) 6:11–21. doi: 10.1007/s11739-010-0407-4, PMID: 20517656

[4] Giustina A Bouillon R Dawson-Hughes B Ebeling PR Lazaretti-Castro M Lips P. Vitamin D in the older population: a consensus statement. Endocrine. (2023) 79:31–44. doi: 10.1007/s12020-022-03208-3, PMID: 36287374 PMC9607753

[5] Durvasula S Kok C Sambrook PN Cumming RG Lord SR March LM. Sunlight and health: attitudes of older people living in intermediate care facilities in southern Australia. Arch Gerontol Geriatr. (2010) 51:e94–9. doi: 10.1016/j.archger.2010.01.008, PMID: 20153063

[6] Grieger JA Nowson CA. Nutrient intake and plate waste from an Australian residential care facility. Euro J Clin Nutr. (2007) 61:655–63. doi: 10.1038/sj.ejcn.1602565, PMID: 17151591

[7] Nowson CA Sherwin AJ McPhee JG Wark JD Flicker L. Energy, protein, calcium, vitamin D and fibre intakes from meals in residential care establishments in Australia. Asia Pac J Clin Nutr. (2003) 12:172–7. 12810407

[8] Akner G Flöistrup H. Individual assessment of intake of energy, nutrients and water in 54 elderly multidiseased nursing-home residents. J Nutr Health Aging. (2003) 7:1–12. 12679834

[9] National Health and Medical Research Council. Nutrient reference values for Australia and New Zealand. Canberra: Commonwealth of Australia (2006).

[10] Williams J Williams C. Responsibility for vitamin D supplementation of elderly care home residents in England: falling through the gap between medicine and food. BMJ Nutr Prev Health. (2020) 3:256–62. doi: 10.1136/bmjnph-2020-000129, PMID: 33521536 PMC7841808

[11] Walker P Kifley A Kurrle SD Cameron ID. Increasing the uptake of vitamin D supplement use in Australian residential aged care facilities: results from the vitamin D implementation (ViDAus) study. BMC Ger. (2020) 20:84. doi: 10.1186/s12877-020-01784-5, PMID: 33023492 PMC7542101

[12] Kupisz-Urbańska M Łukaszkiewicz J Marcinowska-Suchowierska E. Vitamin D in elderly. London, UK: IntechOpen (2021).

[13] Downer S Berkowitz SA Harlan TS Olstad DL Mozaffarian D. Food is medicine: actions to integrate food and nutrition into healthcare. BMJ. (2020) 369:m2482. doi: 10.1136/bmj.m2482, PMID: 32601089 PMC7322667

[14] Berkowitz SA Terranova J Hill C Ajayi T Linsky T Tishler LW. Meal delivery programs reduce the use of costly health care in dually eligible Medicare and Medicaid beneficiaries. Health Aff. (2018) 37:535–42. doi: 10.1377/hlthaff.2017.0999, PMID: 29608345 PMC6324546

[15] Berkowitz SA Terranova J Randall L Cranston K Waters DB Hsu J. Association between receipt of a medically tailored meal program and health care use. JAMA Intern Med. (2019) 179:786–93. doi: 10.1001/jamainternmed.2019.0198, PMID: 31009050 PMC6547148

[16] Seligman HK Lyles C Marshall MB Prendergast K Smith MC Headings A. A pilot food bank intervention featuring diabetes-appropriate food improved glycemic control among clients in three states. Health Aff. (2015) 34:1956–63. doi: 10.1377/hlthaff.2015.0641, PMID: 26526255

[17] Trapl ES Smith S Joshi K Osborne A Benko M Matos AT. Dietary impact of produce prescriptions for patients with hypertension. Prev Chronic Dis. (2018) 15:E138. doi: 10.5888/pcd15.180301, PMID: 30447106 PMC6266424

[18] Hugo C Isenring E Miller M Marshall S. Cost-effectiveness of food, supplement and environmental interventions to address malnutrition in residential aged care: a systematic review. Age Ageing. (2018) 47:356–66. doi: 10.1093/ageing/afx187, PMID: 29315355

[19] IulianoSWoodsJRobbinsJ. Consuming two additional serves of dairy food a day significantly improves energy and nutrient intakes in ambulatory aged care residents: a feasibility study. J Nutr Health Aging. (2013) 17:509–13. doi: 10.1007/s12603-013-0025-823732546

[20] Sossen L Bonham M Porter J. Can fortified, nutrient-dense and enriched foods and drink-based nutrition interventions increase energy and protein intake in residential aged care residents? A systematic review with meta-analyses. Int J Nurs Stud. (2021) 124:104088. doi: 10.1016/j.ijnurstu.2021.104088 34717275

[21] Mak T Louro CS. The role of nutrition in active and healthy ageing – For prevention and treatment of age-related diseases: Evidence so far. EUR 26666. Luxembourg: Publications Office of the European Union (2014).

[22] Lindner-Rabl S Wagner V Matijevic A Herzog C Lampl C Traub J. Clinical interventions to improve nutritional care in older adults and patients in primary healthcare - a scoping review of current practices of health care practitioners. Clin Interv Aging. (2022) 17:1–13. doi: 10.2147/CIA.S343307, PMID: 35023909 PMC8747528

[23] Feeney JM Miller AM Roupas P. Mushrooms-biologically distinct and nutritionally unique: exploring a "third food kingdom". Nutr Today. (2014) 49:301–7. doi: 10.1097/NT.0000000000000063, PMID: 25435595 PMC4244211

[24] Bechraki L van den Heuvel EGHM de Groot LC Groenendijk I. The mutritional benefit of UV-exposed mushrooms for the Dutch population: modeling the addition of UV-exposed mushrooms to the diet. Curr Dev Nutrition. (2023) 7:102039. doi: 10.1016/j.cdnut.2023.102039, PMID: 38162998 PMC10756956

[25] Blumfield M Abbott K Duve E Cassettari T Marshall S Fayet-Moore F. Examining the health effects and bioactive components in Agaricus bisporus mushrooms: a scoping review. J Nutr Biochem. (2020) 84:108453. doi: 10.1016/j.jnutbio.2020.108453, PMID: 32653808

[26] Gallotti F Lavelli V. The effect of UV irradiation on vitamin D2 content and antioxidant and antiglycation activities of mushrooms. Food Secur. (2020) 9:1087. doi: 10.3390/foods9081087, PMID: 32784944 PMC7464819

[27] Malik MA Jan Y Haq A Kaur J Panda BP. Enhancement of vitamin D2 in edible mushroom using ultraviolet irradiation and assessing its storage and cooking stability. Nutr Food Sci. (2022) 52:1254–69. doi: 10.1108/NFS-12-2021-0391

[28] Phillips KM Rasor AS. A nutritionally meaningful increase in vitamin D in retail mushrooms is attainable by exposure to sunlight prior to consumption. J Nutr Food Sci. (2013) 3:1. doi: 10.4172/2155-9600.1000236

[29] Rondanelli M Moroni A Zese M Gasparri C Riva A Petrangolini G. Vitamin D from UV-irradiated mushrooms as a way for vitamin D supplementation: a systematic review on classic and nonclassic effects in human and animal models. Antiox. (2023) 12:736. doi: 10.3390/antiox12030736, PMID: 36978984 PMC10045067

[30] Urbain P Jakobsen J. Dose–response effect of sunlight on vitamin D2 production in Agaricus bisporus mushrooms. J Ag Food Chem. (2015) 63:8156–61. doi: 10.1021/acs.jafc.5b02945, PMID: 26314311

[31] Cardwell G Bornman J James A Black L. A review of mushrooms as a potential source of dietary vitamin D. Nutrients. (2018) 10:1498. doi: 10.3390/nu10101498, PMID: 30322118 PMC6213178

[32] Jiang Q Zhang M Mujumdar AS. UV induced conversion during drying of ergosterol to vitamin D in various mushrooms: effect of different drying conditions. Trends Food Sci Technol. (2020) 105:200–10. doi: 10.1016/j.tifs.2020.09.011, PMID: 32982063 PMC7508054

[33] Starck C Cassettari T Wright J Petocz P Beckett E Fayet-Moore F. Mushrooms: a food-based solution to vitamin D deficiency to include in dietary guidelines. F Nutr. (2024) 11:11. doi: 10.3389/fnut.2024.1384273, PMID: 38660061 PMC11039838

[34] Kleftaki SA Amerikanou C Gioxari A Lantzouraki DZ Sotiroudis G Tsiantas K. A randomized controlled trial on Pleurotus eryngii mushrooms with antioxidant compounds and vitamin D(2) in managing metabolic disorders. Antiox. (2022) 11:113. doi: 10.3390/antiox11112113, PMID: 36358485 PMC9686718

[35] Urbain P Singler F Ihorst G Biesalski HK Bertz H. Bioavailability of vitamin D₂ from UV-B-irradiated button mushrooms in healthy adults deficient in serum 25-hydroxyvitamin D: a randomized controlled trial. Eur J Clin Nutr. (2011) 65:965–71. doi: 10.1038/ejcn.2011.53, PMID: 21540874

[36] Food Standards Australia New Zealand. Australian food composition database - release 2.0. Canberra, Australia: Food Standards Australia New Zealand (FSANZ) (2022).

[37] Das S Prakash B. Edible mushrooms: nutritional composition and medicinal benefits for improvement in quality life In: Prakash B, editor. Research and technological advances in food science. Amsterdam, Netherlands: Elsevier (2022). 269–300. doi: 10.1016/b978-0-12-824369-5.00013-0

[38] Sganzerla WG Todorov SD Da Silva APG. Research trends in the study of edible mushrooms: nutritional properties and health benefits. Int J Med Mush. (2022) 24:1–18. doi: 10.1615/IntJMedMushrooms.2022043738, PMID: 35695585

[39] Niazi AR Ghafoor A. Different ways to exploit mushrooms: a review. All Life. (2021) 14:450–60. doi: 10.1080/26895293.2021.1919570

[40] McArthur C Bai Y Hewston P Giangregorio L Straus S Papaioannou A. Barriers and facilitators to implementing evidence-based guidelines in long-term care: a qualitative evidence synthesis. Imp Sci. (2021) 16:70. doi: 10.1186/s13012-021-01140-0, PMID: 34243789 PMC8267230

[41] Nakamura K Nashimoto M Hori Y Yamamoto M. Serum 25-hydroxyvitamin D concentrations and related dietary factors in peri- and postmenopausal Japanese women. Am J Clin Nutr. (2000) 71:1161–5. doi: 10.1093/ajcn/71.5.1161, PMID: 10799378

[42] Poscia A Milovanovic S La Milia DI Duplaga M Grysztar M Landi F. Effectiveness of nutritional interventions addressed to elderly persons: umbrella systematic review with meta-analysis. Euro J Pub Health. (2018) 28:275–83. doi: 10.1093/eurpub/ckx199, PMID: 29228152

[43] Eldridge SM Chan CL Campbell MJ Bond CM Hopewell S Thabane L. CONSORT 2010 statement: extension to randomised pilot and feasibility trials. BMJ. (2016) 355:i5239. doi: 10.1136/bmj.i5239, PMID: 27777223 PMC5076380

[44] Granic A Hurst C Dismore L Stevenson E Sayer AA Aspray T. Feasibility and acceptability of a milk and resistance exercise intervention to improve muscle function in community-dwelling older adults (MIlkMAN): pilot study. PLoS One. (2020) 15:e0235952. doi: 10.1371/journal.pone.0235952, PMID: 32649708 PMC7351162

[45] Wheeler M Abbey KL Capra SM. Choice on the menu: increasing meal choice for people living in residential aged care, a pilot study. J Hum Nutr Diet. (2025) 38:e13401. doi: 10.1111/jhn.13401, PMID: 39611268

[46] Williams P Walton K. Plate waste in hospitals and strategies for change. Euro J Clin Nutri Metab. (2011) 6:e235–41. doi: 10.1016/j.eclnm.2011.09.006

[47] Subar AF Kirkpatrick SI Mittl B Zimmerman TP Thompson FE Bingley C. The automated self-administered 24-hour dietary recall (ASA24): a resource for researchers, clinicians, and educators from the National Cancer Institute. J Acad Nutr Diet. (2012) 112:1134–7. doi: 10.1016/j.jand.2012.04.016, PMID: 22704899 PMC3721511

[48] Xyris FoodWorks 10 professional, v10.0 [internet]. (2019). Available online at: https://xyris.com.au/ (Accessed July 12, 2024).

[49] NVivo 14 [Internet]. Lumivero Pty ltd. (2023). Available online at: https://lumivero.com/product/nvivo-14/ (Accessed June 10, 2024).

[50] Braun V Clarke V. Using thematic analysis in psychology. Qual Res Psychol. (2006) 3:77–101. doi: 10.1191/1478088706qp063oa

[51] Sandelowski M. Whatever happened to qualitative description? Res Nurs Health. (2000) 23:334–40. doi: 10.1002/1098-240X(200008)23:4<334::AID-NUR9>3.0.CO;2-G 10940958

[52] Neergaard MA Olesen F Andersen RS Sondergaard J. Qualitative description - the poor cousin of health research? BMC Med Res Methodol. (2009) 9:52. doi: 10.1186/1471-2288-9-52, PMID: 19607668 PMC2717117

[53] National Health and Medical Research Council. Nutrient reference values for Australia and New Zealand Canberra: National Health and Medical Research Council, Australian Government Department of Health and ageing, New Zealand ministry of Health; (2006) Available online at: https://www.eatforhealth.gov.au/nutrient-reference-values (Accessed January, 2024).

[54] Fulgoni VL Agarwal S. Nutritional impact of adding a serving of mushrooms on usual intakes and nutrient adequacy using National Health and nutrition examination survey 2011–2016 data. Food Sci Nutr. (2021) 9:1504–11. doi: 10.1002/fsn3.2120 33747464 PMC7958531

[55] Ministry of Health. Competence Center for Climate and Health of Gesundheit Österreich GmbH. Austrian Agency for Health and Food Safety, Austrian society for nutrition In: Austrian nutrition recommendations. Vienna: Ministry of Health (2024)

[56] The German Nutrition Society. DGE nutrition circle. Germany: The German Nutrition Society (2024).

[57] Mehrotra A Calvo MS Beelman RB Levy E Siuty J Kalaras MD. Bioavailability of vitamin D2 from enriched mushrooms in prediabetic adults: a randomized controlled trial. Eur J Clin Nutr. (2014) 68:1154–60. doi: 10.1038/ejcn.2014.157, PMID: 25117997

[58] Stephensen CB Zerofsky M Burnett DJ Lin YP Hammock BD Hall LM. Ergocalciferol from mushrooms or supplements consumed with a standard meal increases 25-hydroxyergocalciferol but decreases 25-hydroxycholecalciferol in the serum of healthy adults. J Nutr. (2012) 142:1246–52. doi: 10.3945/jn.112.159764, PMID: 22623385

[59] Cashman KD Kiely M Seamans KM Urbain P. Effect of ultraviolet light-exposed mushrooms on vitamin D status: liquid chromatography-tandem mass spectrometry reanalysis of biobanked sera from a randomized controlled trial and a systematic review plus meta-analysis. J Nutr. (2016) 146:565–75. doi: 10.3945/jn.115.223784, PMID: 26865648

[60] Pinto JM Merzbach V Willmott AGB Antonio J Roberts J. Assessing the impact of a mushroom-derived food ingredient on vitamin D levels in healthy volunteers. J Int Soc Sports Nutr. (2020) 17:54. doi: 10.1186/s12970-020-00387-0, PMID: 33176826 PMC7659128

[61] Zajac IT Barnes M Cavuoto P Wittert G Noakes M. The effects of vitamin D-enriched mushrooms and vitamin D3 on cognitive performance and mood in healthy elderly adults: a randomised, double-blinded, placebo-controlled trial. Nutrients. (2020) 12:847. doi: 10.3390/nu12123847, PMID: 33339304 PMC7766163

[62] Shanely RA Nieman DC Knab AM Gillitt ND Meaney MP Jin F. Influence of vitamin D mushroom powder supplementation on exercise-induced muscle damage in vitamin D insufficient high school athletes. J Sports Sci. (2014) 32:670–9. doi: 10.1080/02640414.2013.847279, PMID: 24117183

[63] Stepien M O'Mahony L O'Sullivan A Collier J Fraser WD Gibney MJ. Effect of supplementation with vitamin D2-enhanced mushrooms on vitamin D status in healthy adults. J Nutr Sci. (2013) 2:e29. doi: 10.1017/jns.2013.22, PMID: 25191578 PMC4153019

[64] Nieman DC Gillitt ND Shanely RA Dew D Meaney MP Luo B. Vitamin D2 supplementation amplifies eccentric exercise-induced muscle damage in NASCAR pit crew athletes. Nutrients. (2013) 6:63–75. doi: 10.3390/nu6010063, PMID: 24362707 PMC3916849

[65] Lacey LF Armstrong DJ Royle E Magee P Pourshahidi LK Ray S. Cost-effectiveness of vitamin D3 supplementation in older adults with vitamin D deficiency in Ireland. BMJ Nutr Prev Health. (2022) 5:98–105. doi: 10.1136/bmjnph-2021-000382, PMID: 35814728 PMC9237877

[66] Liu RH. Health-promoting components of fruits and vegetables in the diet. Adv Nutr. (2013) 4:384S–92S. doi: 10.3945/an.112.003517, PMID: 23674808 PMC3650511

[67] Chen X Li H Zhang B Deng Z. The synergistic and antagonistic antioxidant interactions of dietary phytochemical combinations. Crit Rev Food Sci Nutr. (2022) 62:5658–77. doi: 10.1080/10408398.2021.1888693, PMID: 33612011

[68] Merrell J Philpin S Warring J Hobby D Gregory V. Addressing the nutritional needs of older people in residential care homes. Health Soc Care Comm. (2012) 20:208–15. doi: 10.1111/j.1365-2524.2011.01033.x, PMID: 21985114

[69] Wang D Everett B Brunero S Northall T Villarosa AR Salamonson Y. Perspectives of residents and staff regarding food choice in residential aged care: a qualitative study. J Clin Nurs. (2020) 29:626–37. doi: 10.1111/jocn.15115, PMID: 31769898

[70] Heaven B Bamford C May C Moynihan P. Adapting menus in care homes to meet the foods standards agency guidelines: a qualitative study of barriers and facilitators to change. Proc Nutr Soc. (2010) 69:OCE6. doi: 10.1017/S002966511000354X, PMID: 40247472

[71] Haryono S Krisanty P Manurung S. Diet health education effect on elderly behavior with hypertension. Asian J App Sci. (2018) 6:5566. doi: 10.24203/ajas.v6i6.5566, PMID: 40330332

[72] Pelletier S Kundrat S Hasler CM. Effects of an educational program on intent to consume functional foods. J Am Diet Assoc. (2002) 102:1297–300. doi: 10.1016/S0002-8223(02)90286-5, PMID: 12792631

[73] Ložnjak P Jakobsen J. Stability of vitamin D3 and vitamin D2 in oil, fish and mushrooms after household cooking. Food Chem. (2018) 254:144–9. doi: 10.1016/j.foodchem.2018.01.182 29548435

[74] Sum S Cao J Hu J Vanous C. The nutrient density of USDA's sample menu measured by the nutrition rich food (NRF) index (OR14-02-19). Curr Dev Nutr. (2019) 3:nzz038.OR14-02-19. doi: 10.1093/cdn/nzz038.OR14-02-19, PMID: 39664488

[75] Whitton C Ramos-García C Kirkpatrick SI Healy JD Dhaliwal SS Boushey CJ. A systematic review examining contributors to misestimation of food and beverage intake based on short-term self-report dietary assessment instruments administered to adults. Adv in Nutr. (2022) 13:2620–65. doi: 10.1093/advances/nmac085, PMID: 36041186 PMC9776649

[76] Gundumogula M. Importance of focus groups in qualitative research. Int J Hum & Soc Stud. (2020) 8:82. doi: 10.24940/theijhss/2020/v8/i11/HS2011-082, PMID: 40346534

[77] Aschbrenner KA Kruse G Gallo JJ Plano Clark VL. Applying mixed methods to pilot feasibility studies to inform intervention trials. Pilot Feasibility Stud. (2022) 8:217. doi: 10.1186/s40814-022-01178-x, PMID: 36163045 PMC9511762

