# Supplementary material for: The mushrooms on the menu (MOM) study: vitamin D mushrooms (UV-exposed) are a feasible and acceptable way to increase vitamin D intake in a residential aged care facility
Source: Front Public Health. 2025 Jun 18;13:1568202. doi: 10.3389/fpubh.2025.1568202 (PMC12213422; doi:10.3389/fpubh.2025.1568202)
Supplement: Supplementary file 1 [file Supplementary_file_1.docx]

**Supplementary Table 1. Standard and MOM recipes**

| Meal name | Meal type | Baseline menu | MOM Menu |
| --- | --- | --- | --- |
| 3 cheese ravioli with garlic cream sauce | Standard | Y | Y |
| Asian vermicelli and prawn salad with caramelized lime | Standard | Y | Y |
| Baked gnocchi and broccoli gratin | Standard | Y | Y |
| Beef tagine | Standard | Y | Y |
| BLT turkey salad | Standard | Y | Y |
| Broccoli and baby beets salad with roasted chickpeas | Standard | Y | Y |
| Broccoli, cheddar and quinoa gratin | Standard | Y | Y |
| Butternut squash soup | Standard | Y | Y |
| Cauliflower soup | Standard | Y | Y |
| Champagne ham and grilled pear salad | Standard | Y | Y |
| Chargrilled honey chicken salad with pickled ginger and cashew nuts | Standard | Y | Y |
| Chargrilled vegetable skewers on saffron rice with sweet and sour dipping sauce | Standard | Y | Y |
| Cheese ravioli carbonara | Standard | Y | Y |
| Chicken and roasted pumpkin risotto | Standard | Y | Y |
| Chicken Caesar salad | Standard | Y | Y |
| Chicken curry and rice (mild) | Standard | Y | Y |
| Chicken parmigiana with chips and salad | Standard | Y | Y |
| Chicken stir-fry and roasted cashew nuts on a bed of steamed rice | Standard | Y | Y |
| Chicken Waldorf dinner bowl | Standard | Y | Y |
| Chorizo and pumpkin salad with roasted pine nuts | Standard | Y | Y |
| Confit Duck in orange glaze | Standard | Y | Y |
| Cream of chicken and sweetcorn soup | Standard | Y | Y |
| Creamy goats cheese polenta with ratatouille vegetables | Standard | Y | Y |
| Creamy honey mustard chicken, with potato bake | Standard | Y | Y |
| Creamy Thai carrot and sweet potato soup | Standard | Y | Y |
| Creamy vegetable soup | Standard | Y | Y |
| Crumbed chicken salad with tomato and avocado salsa | Standard | Y | Y |
| Crumbed chicken schnitzel, with bacon onion potato bake and Diane sauce | Standard | Y | Y |
| Crumbed steak with mushroom sauce and pumpkin mash | Standard | Y | Y |
| Crushed chats with smoked bacon and red lentil salad | Standard | Y | Y |
| Eggplant parmigiana | Standard | Y | Y |
| Fish and chips with homemade tartare sauce | Standard | Y | Y |
| Fisherman’s crumble | Standard | Y | Y |
| French onion soup | Standard | Y | Y |
| Glazed orange and ginger pork steaks with roasted garlic chats | Standard | Y | Y |
| Greek Salad with roasted almonds and grilled halloumi | Standard | Y | Y |
| Grilled fish and parsley sauce | Standard | Y | Y |
| Grilled Tasmanian salmon with saffron rice and cream white sauce | Standard | Y | Y |
| Healthy minestrone soup | Standard | Y | Y |
| Healthy potato and leek soup | Standard | Y | Y |
| Homemade beef lasagne | Standard | Y | Y |
| Lamb stew and dumplings | Standard | Y | Y |
| Meat lovers’ pizza | Standard | Y | Y |
| Mediterranean mixed bean and baby spinach salad with wild rice | Standard | Y | Y |
| Mediterranean pasta salad with smoked chicken | Standard | Y | Y |
| Mild beef Massaman curry with saffron rice and mango salsa | Standard | Y | Y |
| Mixed sandwiches | Standard | Y | Y |
| Moroccan lamb salad | Standard | Y | Y |
| Oriental chicken and corn soup | Standard | Y | Y |
| Oven baked smoked cod with roasted chats and white wine herb sauce | Standard | Y | Y |
| Pasta salad with tuna and herb dressing | Standard | Y | Y |
| Pink Salmon salad with citrus dressing | Standard | Y | Y |
| Pork chop with sauteed apple and roasted potato | Standard | Y | Y |
| Potato and bacon salad with Dijon mustard mayo | Standard | Y | Y |
| Potato and pea salad with smoked trout | Standard | Y | Y |
| Prawn laksa | Standard | Y | Y |
| Pumpkin and chickpea Rogan josh | Standard | Y | Y |
| Red lentil and baby beet salad with balsamic glaze | Standard | Y | Y |
| Red lentil and pumpkin soup | Standard | Y | Y |
| Red lentil and tomato soup | Standard | Y | Y |
| Roast chicken | Standard | Y | Y |
| Roast pork | Standard | Y | Y |
| Roast turkey | Standard | Y | Y |
| Sage and apple pork sausages with potato bake and onion gravy | Standard | Y | Y |
| Salt and pepper calamari with chips and salad | Standard | Y | Y |
| Seafood curry | Standard | Y | Y |
| Seafood plate | Standard | Y | Y |
| Seafood risotto | Standard | Y | Y |
| Seafood salad | Standard | Y | Y |
| Shredded duck salad with honey soy dressing | Standard | Y | Y |
| Silverside salad with baby spinach and tomato relish | Standard | Y | Y |
| Slow cooked Moroccan lamb with mashed potato | Standard | Y | Y |
| Spinach ricotta and mushroom frittata | Standard | Y | Y |
| Stuffed tomato | Standard | Y | Y |
| Sweet and sour pork with steamed rice | Standard | Y | Y |
| Sweet and sour tofu | Standard | Y | Y |
| Tassel salmon fish cakes with lemon grass and ginger | Standard | Y | Y |
| Tennessee pulled pork salad with black beans and corn | Standard | Y | Y |
| Thai beef salad | Standard | Y | Y |
| Tofu stir fry | Standard | Y | Y |
| Tuna niçoise salad | Standard | Y | Y |
| Turkey, avocado and crunchy noodle salad | Standard | Y | Y |
| Vegetarian crumble | Standard | Y | Y |
| Vegetarian pasta bake | Standard | Y | Y |
| Vegetarian risotto | Standard | Y | Y |
| Vegetarian sausages with pumpkin mash and mustard and chive sauce | Standard | Y | Y |
| Vegetarian savory crepe | Standard | Y | Y |
| Vegetarian savory muffin with saffron rice and sour cream and chives | Standard | Y | Y |
| Veggie hotpot | Standard | Y | Y |
| Veggie looks like chicken strips with satay and rice | Standard | Y | Y |
| Veggie patties | Standard | Y | Y |
| Veggie pizza | Standard | Y | Y |
| Veggie Tender schnitzel with potato bake | Standard | Y | Y |
| Waldorf salad | Standard | Y | Y |
| Beef and bacon red wine stew | Standard | Y | N |
| Beef and red wine sausages on pesto mash with thick gravy | Standard | Y | N |
| Beef moussaka | Standard | Y | N |
| Beef stroganoff and rice | Standard | Y | N |
| Braised lamb shank served with leek and shallot mash hotpot | Standard | Y | N |
| Chicken a la king with puff pastry case | Standard | Y | N |
| Chicken and mushroom crepe with roasted pumpkin mash | Standard | Y | N |
| Chicken breast with spinach cream sauce | Standard | Y | N |
| Chicken Casserole with roasted root veggies | Standard | Y | N |
| Chicken ratatouille pasta bake | Standard | Y | N |
| Cream of mushroom soup | Standard | Y | N |
| Creamy mushroom carbonara | Standard | Y | N |
| Diane meatloaf with French onion mash and red wine gravy | Standard | Y | N |
| Ham and split pea soup | Standard | Y | N |
| Homemade beef cottage pie | Standard | Y | N |
| Homemade chicken and mushroom pie with sweet potato wedges | Standard | Y | N |
| Penne Bolognese with shredded parmesan | Standard | Y | N |
| Red lentil and walnut bolognaise with tagliatelle pasta | Standard | Y | N |
| Ricotta and spinach tartlet with caramelized red onion | Standard | Y | N |
| Roast beef | Standard | Y | N |
| Roast lamb | Standard | Y | N |
| Sausage casserole | Standard | Y | N |
| Shoreline vegetable stack with basil and tomato sauce | Standard | Y | N |
| Slow cooked lab ragu and gnocchi | Standard | Y | N |
| Spinach and ricotta ravioli served with a rich Italian tomato and basil sauce | Standard | Y | N |
| Spinach, bacon and parmesan quiche with sweet potato roti | Standard | Y | N |
| Vegetarian lasagne | Standard | Y | N |
| Vegetarian stuffed capsicum | Standard | Y | N |
| Bacon and mushroom quiche with sweet potato roti | Vitamin D mushroom | N | Y |
| Beef and bacon red wine stew | Vitamin D mushroom | N | Y |
| Beef cottage pie | Vitamin D mushroom | N | Y |
| Beef moussaka | Vitamin D mushroom | N | Y |
| Beef stroganoff and rice | Vitamin D mushroom | N | Y |
| Braised lamb and mushroom hotpot | Vitamin D mushroom | N | Y |
| Chicken and mushroom crepe with roasted pumpkin mash | Vitamin D mushroom | N | Y |
| Chicken breast with spinach cream sauce and roasted mushrooms | Vitamin D mushroom | N | Y |
| Chicken casserole | Vitamin D mushroom | N | Y |
| Chicken in white wine sauce with puff pastry vol-vent | Vitamin D mushroom | N | Y |
| Chicken ratatouille pasta bake | Vitamin D mushroom | N | Y |
| Cream of mushroom soup | Vitamin D mushroom | N | Y |
| Creamy mushroom carbonara | Vitamin D mushroom | N | Y |
| Diane meatloaf with French onion mash and red wine gravy | Vitamin D mushroom | N | Y |
| Grilled fish and parsley sauce | Vitamin D mushroom | N | Y |
| Homemade chicken and mushroom pie with sweet potato wedges | Vitamin D mushroom | N | Y |
| Mushroom curry | Vitamin D mushroom | N | Y |
| Mushroom strudel | Vitamin D mushroom | N | Y |
| Penne pasta Bolognese with shredded parmesan | Vitamin D mushroom | N | Y |
| Roast beef | Vitamin D mushroom | N | Y |
| Roast lamb | Vitamin D mushroom | N | Y |
| Sausage casserole | Vitamin D mushroom | N | Y |
| Sausages and mash with mushroom sauce | Vitamin D mushroom | N | Y |
| Shoreline vegetable stack with basil and tomato sauce | Vitamin D mushroom | N | Y |
| Slow cooked lamb ragu with mushroom and gnocchi | Vitamin D mushroom | N | Y |
| Spinach and ricotta ravioli with rich Italian tomato and basil sauce with mushrooms | Vitamin D mushroom | N | Y |
| Steak and mushroom pie with roasted pumpkin mash | Vitamin D mushroom | N | Y |
| Vegetarian lasagne | Vitamin D mushroom | N | Y |

**Supplementary Table 2: Frequency of meal portion consumption among RC participants during baseline and MOM phases**

| **Study phase** | **Frequency of meal portion consumption** | | | | | | |  |  | **t test** |
| --- | --- | --- | --- | --- | --- | --- | --- | --- | --- | --- |
|  | **0%** | **10%** | **25%** | **50%** | **75%** | **90%** | **100%** | **Mean (±SD)** | **CI** | ***p* (2-tailed)** |
| Standard Menu | 5 | 23 | 32 | 61 | 100 | 123 | 191 | 0.8 (**±**0.3) | 0.8-0.8 | 0.08 |
| MOM | 21 | 15 | 47 | 68 | 94 | 109 | 199 | 0.8 (**±**0.3) | 0.7-0.8 |  |

SD, standard deviation; CI, confidence interval; SE, standard error

**Supplementary Table 3. Changes in mean micronutrient intakes of RC and IL participants from baseline to MOM phase**

| **Nutrient** | **Standard Menu** |  | **MOM** |  | **t test** |
| --- | --- | --- | --- | --- | --- |
|  | **Mean per meal (±SD)** | **CI** | **Mean per meal (±SD)** | **CI** | ***p* (2-tailed)** |
| **Residential care participants** | |  |  |  |  |
| Vitamin A eq. (µg) | 128 (±427.4) | 1170.0-1390.8 | 1453 (±441.2) | 1338-1567 | 0.0001 |
| Vitamin C (mg) | 236.3 (±92.2) | 212.4-260.1 | 305.4 (±99.1) | 279.8-331.0 | <0.0001 |
| Vitamin E (mg) | 9.3 (±2.5) | 8.7-10.0 | 8.6 (±2.2) | 8.0-9.1 | 0.02 |
| Thiamine (mg) | 0.6 (±0.2) | 0.6-0.7 | 0.6 (±0.2) | 0.5-0.6 | 0.01 |
| Riboflavin (mg) | 0.6 (±0.1) | 0.5-0.6 | 0.6 (±0.1) | 0.5-0.6 | 0.8 |
| Niacin eq. (mg) | 23.0 (±6.4) | 21.3-24.6 | 24.8 (±6.5) | 23.1-26.5 | 0.007 |
| Vitamin B6 (mg) | 1.6 (±0.6) | 1.5-1.8 | 1.6 (±0.5) | 1.5-1.7 | 0.7 |
| Folate DFE (µg) | 232.0 (±62.2) | 215.9-248.1 | 244.7 (±63.4) | 228.3-261.1 | 0.03 |
| Vitamin B12 (µg) | 1.9 (±0.7) | 1.7-2.1 | 2.1 (±0.8) | 1.8-2.3 | 0.1 |
| Calcium (mg) | 240.2 (62.9) | 224.0-256.5 | 262.4 (±88.7) | 239.5-285.3 | 0.03 |
| Iron (mg) | 5.8 (±1.4) | 5.4-6.1 | 6.0 (±1.5) | 5.6-6.4 | 0.2 |
| Magnesium (mg) | 145.6 (±30.6) | 137.7-153.5 | 148.5 (±33.7) | 139.8-157.2 | 0.4 |
| Phosphorous (mg) | 703.4 (±160.0) | 662.2-744.7 | 748.4 (±175.0) | 703.2-793.6 | 0.01 |
| Potassium (mg) | 2020.3 (±522.4) | 1885.3-2155 | 2159 (±570.0) | 2012.-2306 | 0.03 |
| Selenium (µg) | 50.3 (±13.6) | 46.8-53.8 | 49.7 (±13.1) | 46.3-53.1 | 0.8 |
| Zinc (mg) | 5.4 (±1.5) | 5.0-5.7 | 5.4 (±1.6) | 5.0-5.8 | 0.8 |
| **Independent living participants** | |  |  |  |  |
| Vitamin A eq. (µg) | 1417.4 (±1134) | 655.3-2180 | 1119 (±932.3) | 492.5-1745 | 0.5 |
| Vitamin C (mg) | 165.1 (±102.8) | 96.0-234.2 | 137.8 (±89.7) | 77.5-198.1 | 0.5 |
| Vitamin E (mg) | 12.5 (±5.4) | 8.8-16.1 | 10.6 (±3.9) | 8.1-13.2 | 0.4 |
| Thiamine (mg) | 1.4 (±0.4) | 1.1-1.7 | 1.3 (±0.4) | 1.0-1.7 | 0.9 |
| Riboflavin (mg) | 2.0 (±0.7) | 1.5-2.4 | 1.8 (±0.5) | 1.5-2.1 | 0.5 |
| Niacin eq. (mg) | 25.5 (±10.6) | 18.4-32.7 | 26.0 (±7.2) | 21.1-30.9 | 0.9 |
| Vitamin B6 (mg) | 1.5 (±0.7) | 1.0-2.0 | 1.2 (±0.3) | 1.0-1.4 | 0.2 |
| Folate DFE (µg) | 565.4 (±151.9) | 463.3-667.5 | 616.8 (±155.6) | 512.2-721.4 | 0.4 |
| Vitamin B12 (µg) | 4.3 (±1.7) | 3.2-5.5 | 4.8 (±1.8) | 3.5-6.0 | 0.6 |
| Calcium (mg) | 912.8 (±313.0) | 702.5-1123 | 819.8 (±340.4) | 591.1-1049 | 0.5 |
| Iron (mg) | 11.8 (±3.4) | 9.5-14.1 | 11.8 (±3.3) | 9.6-14.1 | 1.0 |
| Magnesium (mg) | 397.8 (±135.4) | 306.8-488.8 | 372.7 (±109.2) | 299.4-446 | 0.6 |
| Phosphorous (mg) | 1603 (±591.6) | 1206-2001 | 1537.5 (±430.5) | 1248-1827 | 0.8 |
| Potassium (mg) | 3480 (±1193) | 2678.8-4282 | 3609.5 (±1199) | 2804-4415 | 0.8 |
| Selenium (µg) | 98.5 (±41.7) | 70.5-126.5 | 104.6 (±32.3) | 82.9-126.3 | 0.7 |
| Zinc (mg) | 11.3 (±3.6) | 8.8-13.7 | 11.2 (±3.4) | 9.0-13.5 | 1.0 |

RC participants n = 60; IL participants n = 11; SD, standard deviation; CI, confidence intervals; DFE, dietary folate equivalents.

**Supplementary Table 4. MOM post-meal outcome surveys for RC and IL participants on experiences with vitamin D mushroom meals**

| Survey question | n | % |
| --- | --- | --- |
| Residential care participants |  |  |
| Taste of mushroom meal^*^ |  |  |
| Excellent | 54 | 21.6 |
| Good | 141 | 56.4 |
| Average | 42 | 16.8 |
| Poor | 11 | 4.4 |
| Very poor | 2 | 0.01 |
| Presentation of mushroom meal^*^ |  |  |
| Excellent | 34 | 13.6 |
| Good | 151 | 60.4 |
| Average | 53 | 21.2 |
| Poor | 10 | 4.0 |
| Very poor | 2 | 0.01 |
| Overall enjoyment of mushroom meal^*^ |  |  |
| Excellent | 44 | 17.6 |
| Good | 151 | 60.4 |
| Average | 43 | 17.2 |
| Poor | 10 | 4.0 |
| Very poor | 2 | 0.01 |
| Would you order this mushroom meal again?^#^ |  |  |
| Yes | 164 | 66.1 |
| No | 18 | 7.3 |
| Unsure | 66 | 26.6 |
| Independent living participants |  |  |
| Meal occasion when you ate your mushroom meal |  |  |
| Breakfast | 11 | 31.4 |
| Lunch | 4 | 11.4 |
| Dinner | 20 | 57.1 |
| Taste of mushroom meal |  |  |
| Excellent | 29 | 82.9 |
| Good | 6 | 17.1 |
| Average or below | 0 | 0.0 |
| Presentation of mushroom meal |  |  |
| Excellent | 24 | 68.6 |
| Good | 11 | 31.4 |
| Average or below | 0 | 0.0 |
| Texture of mushroom meal |  |  |
| Excellent | 27 | 77.1 |
| Good | 8 | 22.9 |
| Average or below | 0 | 0.0 |
| Overall enjoyment of mushroom meal |  |  |
| Excellent | 29 | 82.9 |
| Good | 5 | 14.3 |
| Average or below | 1 | 2.9 |
| Would you cook this mushroom meal again? |  |  |
| Yes | 35 | 100.0 |
| No | 0 | 0.0 |
| Did you notice any side effect when eating the mushroom meals? |  |  |
| Yes | 0 | 0.0 |
| No | 35 | 100.0 |

RC- residential care; IL-Independent Living. RC response counts vary due to incomplete answers: ^*^250, ^#^248; Number of IL responses: 35

**Supplementary Table 5. Post-MOM phase staff experiences and feasibility of the vitamin D mushroom menu**

| Survey question  (n = 12) | n | % |
| --- | --- | --- |
| Area of work at The Shoreline RAC |  |  |
| Companion | 7 | 63.6 |
| Housekeeping | 1 | 9.0 |
| Food service & kitchen | 2 | 18.2 |
| Other | 1 | 9.0 |
| UV-exposed mushroom meals are easy to prepare |  |  |
| Strongly agree | 0 | 0.0 |
| Agree | 3 | 27.3 |
| Neither agree nor disagree | 1 | 9.0 |
| Disagree or strongly disagree | 0 | 0.0 |
| Not applicable | 8 | 72.7 |
| UV-exposed mushroom meal preparation equipment available |  |  |
| Strongly agree | 1 | 9.0 |
| Agree | 2 | 18.2 |
| Neither agree nor disagree | 1 | 9.0 |
| Disagree or strongly disagree | 0 | 0.0 |
| Not applicable | 7 | 63.6 |
| Adequately trained to prepare UV-exposed mushroom meals |  |  |
| Strongly agree | 2 | 18.2 |
| Agree | 2 | 18.2 |
| Neither agree nor disagree | 0 | 0.0 |
| Disagree or strongly disagree | 0 | 0.0 |
| Not applicable | 7 | 63.6 |
| Adequate time to prepare UV-exposed mushroom meals |  |  |
| Strongly agree | 1 | 9.0 |
| Agree | 2 | 18.2 |
| Neither agree nor disagree | 0 | 0.0 |
| Disagree or strongly disagree | 1 | 9.0 |
| Not applicable | 7 | 63.6 |
| Sufficient administrative support to prepare UV-exposed mushroom meals |  |  |
| Strongly agree | 0 | 0.0 |
| Agree | 4 | 36.4 |
| Neither agree nor disagree | 1 | 9.0 |
| Disagree or strongly disagree | 1 | 9.0 |
| Not applicable | 5 | 45.5 |
| Adequate cold storage for fresh UV-exposed mushrooms |  |  |
| Strongly agree | 1 | 9.0 |
| Agree | 3 | 27.3 |
| Neither agree nor disagree | 0 | 0.0 |
| Disagree or strongly disagree | 0 | 0.0 |
| Not applicable | 7 | 63.6 |
| Support inclusion of UV-exposed mushroom meals in aged care menu’s |  |  |
| Strongly agree | 6 | 54.5 |
| Agree | 5 | 45.5 |
| Neither agree nor disagree | 0 | 0.0 |
| Disagree or strongly disagree | 0 | 0.0 |
| Not applicable | 0 | 0.0 |
| Encouraged residents to order UV-exposed mushroom meals |  |  |
| Strongly agree | 5 | 45.5 |
| Agree | 6 | 54.5 |
| Neither agree nor disagree | 0 | 0.0 |
| Disagree or strongly disagree | 0 | 0.0 |
| Not applicable | 0 | 0.0 |
| Understands the health benefits of UV-exposed mushrooms |  |  |
| Strongly agree | 6 | 54.5 |
| Agree | 5 | 45.5 |
| Neither agree nor disagree | 0 | 0.0 |
| Disagree or strongly disagree | 0 | 0.0 |
| Not applicable | 0 | 0.0 |
| Own mushroom consumption has increased |  |  |
| Strongly agree | 4 | 36.4 |
| Agree | 3 | 27.3 |
| Neither agree nor disagree | 3 | 27.3 |
| Disagree or strongly disagree | 1 | 9.0 |
| Not applicable | 0 | 0.0 |
| Eating UV-exposed mushrooms is a suitable alternative to vitamin D supplementation |  |  |
| Strongly agree | 4 | 36.4 |
| Agree | 3 | 27.3 |
| Neither agree nor disagree | 3 | 27.3 |
| Disagree or strongly disagree | 1 | 9.0 |
| Not applicable | 0 | 0.0 |
| UV-exposed mushroom rich meals can improve health |  |  |
| Strongly agree | 5 | 45.5 |
| Agree | 5 | 45.5 |
| Neither agree nor disagree | 1 | 9.0 |
| Disagree or strongly disagree | 0 | 0.0 |
| Not applicable | 0 | 0.0 |
| I’d like the RAC facility to continue offering the UV- exposed mushroom menu |  |  |
| Strongly agree | 2 | 18.2 |
| Agree | 6 | 54.5 |
| Neither agree nor disagree | 2 | 18.2 |
| Disagree or strongly disagree | 0 | 0.0 |
| Not applicable | 1 | 9.0 |
|  |  |  |

**Supplementary Table 6. Considerations when including vitamin D mushroom meals on a residential aged care facility menu.**

| **Variety** |
| --- |
| - Residents want variety in the way they consume mushrooms and prefer a combination of meals that showcase mushrooms, and other where mushrooms are a complimentary ingredient. |
| - Ongoing daily consumption of meals that heavily showcase mushrooms was not preferred. |
| **Eating occasion frequency** |
| - Residents enjoyed consuming mushrooms at lunch and dinner eating occasions. |
| - Some residents reported consuming mushrooms at breakfast less enjoyable than other eating occasions due to meals showcasing, rather than mushrooms being a complimentary ingredient. |
| - Frequency of mushroom consumption was greater than residents’ perception due to mushrooms being used as a complimentary ingredient, and residents’ lack of awareness that meals contained mushrooms. |
| **Preparation preferences** |
| - Some residents expressed an emotional connection to mushrooms as they reminded them of their childhood, or a food they had long enjoyed eating. |
| - Many residents wanted mushrooms prepared according to their preferences in traditional ways such as with “butter and milk”, and with “sauce”, or “gravy”. Residents disliked when mushrooms were dry or served without a condiment that enhanced the mushrooms texture and/or flavour. |
| - Residents expressed a desire for larger types of mushrooms (such as flat, field or Portobello mushrooms) in addition to the smaller button mushroom varieties. |
| - It was noted that the mushroom soup was a popular meal amongst residents, and the schnitzel with mushroom sauce amongst staff. |
| - Independent living residents enjoyed using being creative incorporating mushrooms in different ways. The only challenge for some was forgetting to collect their UV-mushrooms from the aged care facility kitchen. |
| **Knowledge on mushrooms** |
| - Implementing the study generated interest, awareness and increased resident and staff knowledge on mushrooms. |
| - Knowledge around mushroom preparation, cooking practices and health benefits of UV-exposure were reported. |
| - Residents intended to continue to eat mushrooms after the trial, particularly if they were prepared in accordance with our findings (i.e. with adequate variety, and with eating occasion frequency and preparation preferences considered). |
| - The aged care facility intended to continue purchasing UV-exposed mushrooms after the trial and were investigating the logistics of doing so. |
| **Strategic menu planning** |
| - Staff require low-burden processes to effectively implement a mushroom menu. Clinical and food service staff were unable to take on additional responsibilities to their usual role to facilitate ordering of mushrooms and confirm consumption. |
| - There were various practical challenges to implementing the mushroom menu including: - Balancing the flavour of meals; Mushrooms displaced other ingredients, such as meat, to incorporate an adequate serving size (75g) of UV-exposed mushrooms in each meal, whilst also maintaining acceptable-sized meal portions, as aged care residents often prefer smaller meal portions (≤150g). - Keeping staff burden to implement low; An additional step of weighing mushrooms for each meal prepared by kitchen staff was required. - Forecasting quantity and cost of raw ingredients; Forecasting the quantity of UV-exposed mushrooms to purchase when residents have the option to order mushrooms meals at all eating occasions everyday (and have the option to change pre-determined orders on the day). - An extensive menu required additional planning; Developing a simple mushroom menu with limited mushroom meal options at limited eating occasions (e.g. ≤28 weekly meal options) rather than developing an extensive mushroom menu that includes many mushroom meal options at multiple eating occasions (>50 weekly meal options) required additional planning. |
| - The cost of implementing a mushroom menu exceeded estimations as residents ordered more meals than expected. Offering mushroom meals at multiple eating occasions everyday may increase the risk of costs exceeding budget estimations. |
| - Initial monitoring and evaluation of meal ordering data is recommended to confirm feasibility at specific facility. |

**Supplementary Table 7. Cost per serve of standard menu meals versus MOM meals with vitamin D mushrooms**

| **Standard menu meals** | **Cost per serve** | **MOM meals**  **(include 75 g of vitamin D mushrooms)** | **Cost per serve** | **Cost difference** |
| --- | --- | --- | --- | --- |
| Spinach, bacon and parmesan quiche, sweet potato rosti | $3.45 | Bacon and mushroom quiche, sweet potato rosti | $3.45 | $0.00 |
| Beef and red wine sausages on pesto mash, thick gravy | $4.00 | Beef and red wine sausages, mushroom sauce | $5.05 | $1.05 |
| Beef Bourguignon | $3.90 | Beef Bourguignon | $4.95 | $1.05 |
| Homemade beef cottage pie | $2.95 | Beef cottage pie | $3.75 | $0.80 |
| Beef moussaka | $3.00 | Beef moussaka | $3.50 | $0.50 |
| Beef stroganoff and rice | $4.40 | Beef stroganoff and rice | $4.40 | $0.00 |
| braised lamb shank served with leek and shallot mash hotpot | $3.49 | Braised lamb and mushroom hotpot | $3.59 | $0.10 |
| Chicken and mushroom crepe, roasted pumpkin mash | $3.15 | Chicken and mushroom crepe, roasted pumpkin mash | $3.65 | $0.50 |
| Chicken breast, spinach cream sauce | $3.40 | Chicken breast, spinach cream sauce, roasted mushrooms | $4.45 | $1.05 |
| Chicken casserole with roasted root veggies | $3.20 | Chicken casserole | $3.70 | $0.50 |
| Chicken a la king with puff pastry case | $3.45 | Chicken in white wine sauce, puff pastry vol-vent | $4.50 | $1.05 |
| Chicken ratatouille pasta bake | $4.15 | Chicken ratatouille pasta bake | $5.20 | $1.05 |
| Diane meatloaf with french onion mash and red wine gravy | $3.05 | Diane meatloaf, french onion mash, red wine gravy | $3.45 | $0.40 |
| Grilled fish and parsley sauce | $5.95 | Grilled fish and parsley sauce | $6.45 | $0.50 |
| Homemade chicken and mushroom pie, sweet potato wedges | $3.15 | Homemade chicken and mushroom pie, sweet potato wedges | $3.65 | $0.50 |
| Homemade shepherd’s pie, cheesy sweet potato mash crust | $4.00 | Homemade shepherd’s pie, cheesy sweet potato mash crust | $4.45 | $0.45 |
| Creamy mushroom carbonara | $3.00 | Mushroom carbonara | $3.00 | $0.00 |
| Tagliatelle Bolognese with shredded parmesan | $3.20 | Penne pasta Bolognese with shredded parmesan | $3.70 | $0.50 |
| Roast beef | $3.50 | Roast beef | $3.70 | $0.20 |
| Roast lamb | $3.49 | Roast lamb | $4.54 | $1.05 |
| Sausage casserole | $3.15 | Sausage casserole | $3.45 | $0.30 |
| Shoreline vegetable stack with basil and tomato sauce | $2.70 | Shoreline vegetable stack with basil and tomato sauce | $3.58 | $0.88 |
| Slow cooked lab ragu and gnocchi | $6.48 | Slow cooked lamb ragu with mushroom and gnocchi | $6.98 | $0.50 |
| Spinach and ricotta ravioli served, rich Italian tomato and basil sauce | $2.55 | Spinach and ricotta ravioli, rich Italian tomato and basil sauce, sauteed mushrooms | $3.60 | $1.05 |
| Steak and mushroom pie with roasted pumpkin mash | $4.00 | Steak and mushroom pie with roasted pumpkin mash | $4.00 | $0.00 |
| Vegetarian lasagne | $2.90 | Vegetarian lasagna | $3.20 | $0.30 |
| Mean cost per meal | $3.35 |  | $3.86 | $0.55 |

n = 26 meals adapted to include 75 g of vitamin D mushrooms

**Supplementary Material**

**Appendix 1.**

## Focus group discussion session outline (30 mins)

The following outline will be used to guide focus group discussion sessions.

**Welcome**

- Introduce moderator and assistant
- My role as moderator will be to guide the discussion

**Purpose**

- The purpose of discussion. To understand more about your experiences over the past month implementing the new mushroom menu. We’re having discussions like this with every staff member who has agreed to participate in the MOM study.
- The results will be used to evaluate if implementing a mushroom menu in a residential aged care facility is indeed feasible.

**Guidelines**

- No right or wrong answers, only differing points of view. Please feel free to share your point of view even if it differs from what others have said. Keep in mind that we’re just as interested in negative comments as positive comments, and at times the negative comments are the most helpful.
- You don’t need to agree with others, but you must listen respectfully as others share their views. Please be mindful to allow only one person to speak at a time.
- You may have noticed the recording device. We’re tape recording the session because we don’t want to miss any of your comments. People often say very helpful things in these discussions and we can’t write fast enough to get them all down.
- We’re on a first name basis today and we won’t use any names in our reports. You may be assured of complete confidentiality.
- Rules for mobile phones and pagers. Please turn off, or if you cannot and if you must respond, please do so as quietly as possible and re-join the discussion as soon as you can.

**Open-ended questions – food services group/s**

- Well, let’s begin. Let’s find out some more about each other by going around the table. Please share with us your name and role you had during the MOM study.
- Overall, what did you think of the MOM study?
- We’d like to hear about your experiences in terms of the *feasibility* (practicality) of implementing the mushroom menu. What worked well, what didn’t work so well, what could have been better?
- What did you think about the RAC facilities’ capacity to support the implementation of the mushroom menu? (e.g., Were there any challenges in terms of admin capacity, equipment, expertise, skills, time or budget to implement the mushroom menu?)
- How well do you think you were supported during the implementation of the mushroom menu?
- Were there any challenges for staff complying with implementing the mushroom menu or any other components of the MOM study? Absence from work, absence from training
- Did you notice any change in how the meals were served/presented or given to the residents?
- How did you find the MOM study impacted your own behaviour? (e.g., have you changed your cooking habits, serving size, or learnt more about mushrooms in the workplace/home, have you made any changes to consumption of mushrooms at home?)
- What do you know about mushrooms that you didn’t know before?
- How did you feel about the training and education that was provided to support staff in implementing the MOM study? How well did you feel you were trained and informed to educate residents on the study?
- That brings us to the end of the focus group. Before we wrap up was there anything else that anyone wanted to share? Any final thoughts, reflections, learnings, or comments?

**Open-ended questions – clinical staff group/s**

- Well, let’s begin. Let’s find out some more about each other by going around the table. Please share with us your name and role you had during the MOM study.
- Overall, what did you think of the MOM study?
- We’d like to hear about your experiences in terms of the *feasibility* (practicality) of implementing the mushroom menu. What worked well, what didn’t work so well, what could have been better?
- What did you think about the RAC facilities’ capacity to support the implementation of the mushroom menu? (e.g., Were there any challenges in terms of admin capacity, equipment, expertise, skills, time or budget to implement the mushroom menu?)
- To what extend do you think you were supported during the implementation of the mushroom menu?
- Did you notice any change to the way you cared for the resident during this time e.g., gave more education, assistance in eating or choosing the mushroom meal?
- Were there any challenges for staff complying with implementing the mushroom menu or any other components of the MOM study? E.g., absence from education, absence from work, or bias towards mushrooms!
- How did you find the MOM study impacted your own behaviour? (e.g., have you changed your cooking habits, serving size, or learnt more about mushrooms in the workplace/home, change to consumption of mushrooms at home?)
- What do you know about mushrooms that you didn’t know before?
- How did you feel about the training and education that was provided to support staff in implementing the MOM study? How well did you feel you were trained and informed to educate residents on the study? Is there anything you think needed changing/improving?
- That brings us to the end of the focus group. Before we wrap up was there anything else that anyone wanted to share? Any final thoughts, reflections, learnings, or comments?

**Open-ended questions – participant group/s**

- Well, let’s begin. Let’s find out some more about each other by going around the table. Please share with us your name and little bit about yourself.
- Overall, what did you think of the MOM study?
- We’d like to hear about your experiences eating the meals from the new mushroom menu. What did you like, what didn’t you like, what could have been better?
- What have you learnt about mushrooms?
- How did you feel participating in the MOM study? Did you notice any changes in health?
- Did you find the MOM study changed your own behaviour?
- Would you like Shoreline to keep offering UV-exposed mushroom meals on the menu? Explain why/why not.
- That brings us to the end of the focus group. Before we wrap up was there anything else that anyone wanted to share? Any final thoughts, reflections, learnings, or comments?

**Additional questions**

- Did you noticed any side effects when eating the mushroom meal?
- Did you notice any side effects after eating the mushroom meal?
- Would you like The Shoreline to continue to offer mushrooms on the menu?

**Closing**

Thank you all for your time today. We really appreciate each of you sharing your experiences with the MOM study and contributing to our research.
